# A novel UBE2T inhibitor suppresses Wnt/β-catenin signaling hyperactivation and gastric cancer progression by blocking RACK1 ubiquitination

**DOI:** 10.1038/s41388-020-01572-w

**Published:** 2020-12-15

**Authors:** Zeyuan Yu, Xiangyan Jiang, Long Qin, Haixiao Deng, Jianli Wang, Wen Ren, Hongbin Li, Lei Zhao, Huanxiang Liu, Hong Yan, Wengui Shi, Qi Wang, Changjiang Luo, Bo Long, Huinian Zhou, Hui Sun, Zuoyi Jiao

**Affiliations:** 1 Department of General Surgery, Lanzhou University Second Hospital, 730000 Lanzhou, Gansu China; 2 Cui-ying Experimental Center, Lanzhou University Second Hospital, 730000 Lanzhou, Gansu China; 3 School of pharmacy, Lanzhou University, 730000 Lanzhou, Gansu China; 4 Department of Pathology, Lanzhou University Second Hospital, 730000 Lanzhou, Gansu China; 5 Cancer Center, Beth Israel Deaconess Medical Center, Harvard Medical School, Boston, MA 02215 USA

**Keywords:** Drug development, Gastric cancer

## Abstract

Dysregulation of the Wnt/β-catenin signaling pathway is critically involved in gastric cancer (GC) progression. However, current Wnt pathway inhibitors being studied in preclinical or clinical settings for other cancers such as colorectal and pancreatic cancers are either too cytotoxic or insufficiently efficacious for GC. Thus, we screened new potent targets from β-catenin destruction complex associated with GC progression from clinical samples, and found that scaffolding protein RACK1 deficiency plays a significant role in GC progression, but not APC, AXIN, and GSK3β. Then, we identified its upstream regulator UBE2T which promotes GC progression via hyperactivating the Wnt/β-catenin signaling pathway through the ubiquitination and degradation of RACK1 at the lysine K172, K225, and K257 residues independent of an E3 ligase. Indeed, UBE2T protein level is negatively associated with prognosis in GC patients, suggesting that UBE2T is a promising target for GC therapy. Furthermore, we identified a novel UBE2T inhibitor, M435-1279, and suggested that M435-1279 acts inhibit the Wnt/β-catenin signaling pathway hyperactivation through blocking UBE2T-mediated degradation of RACK1, resulting in suppression of GC progression with lower cytotoxicity in the meantime. Overall, we found that increased UBE2T levels promote GC progression via the ubiquitination of RACK1 and identified a novel potent inhibitor providing a balance between growth inhibition and cytotoxicity as well, which offer a new opportunity for the specific GC patients with aberrant Wnt/β-catenin signaling.

## Introduction

As one of the most globally prevalent cancers, gastric cancer (GC) has an incidence in the world ranking fifth and mortality ranking third in all kinds of cancers according to “National Cancer Report 2019” published by the National Cancer Center [1]. Despite significant progress that has been achieved during the past decade, the improvement of long-term survival for advanced GC patients remains unsatisfactory. The 5-year survival rate of GC patients varies from 30 to 50% after radical gastrectomy combined with chemotherapy [2, 3]. For unresectable GC patients treated with chemotherapy plus trastuzumab, the media survival time could be prolonged from 11.1 to 13.8 months compared with chemotherapy alone [4]. However, the positive rate of erbB2 is only about 20% in GC patients [5, 6]. Therefore, it is urgent to identify new therapeutic targets and more effective drugs to further improve the long-term survival of GC patients.

The Wnt/β-catenin signaling pathway is one of the key cascades that is essential to many physiological processes, such as cell proliferation and differentiation, stem cell renewal, embryogenesis, and tissue homeostasis [7, 8]. Genetic or epigenetic events leading to aberrant Wnt/β-catenin signaling have been associated with different types of human cancers such as colorectal cancer and breast cancer [9–11]. Previous studies also suggested that pathologic disorder of Wnt pathway components could be implicated in GC progression [12]. Members of the Wnt family protein, such as Wnt1, Wnt-2, Wnt2B, Wnt5A, Wnt6, and Wnt10A have been shown to be significantly enhanced in GC [12, 13]. Moreover, loss-of-function mutations or downregulation of Wnt inhibitors such as APC and AXIN1 may also be involved in the tumorigenesis of GC [14–16]. The remarkable event of Wnt/β-catenin signaling pathway activation is that β-catenin translocates into the nucleus due to the loss function of the destruction complex. The receptor for activated protein kinase C (RACK1), as a key scaffold protein, is involved in stabilizing the β-catenin destruction complex [17]. Interestingly, previous studies showed that loss of RACK1 could abolish the stability of the β-catenin destruction complex, resulting in the translocation of β-catenin into the nucleus and gastric carcinogenesis [17]. Thus, it is promising to explore a new therapeutic strategy for GC based on the regulation mechanism of RACK1 on the Wnt/β-catenin signaling pathway.

In this study, we found that current Wnt pathway inhibitors in preclinical or clinical trials for other cancers are either of high cytotoxicity or low efficiency for GC cells. Then, we found a critical ubiquitin-conjugating enzyme E2T (UBE2T) that catalyzes the proteasomal degradation of RACK1 and then induces hyperactivation of Wnt/β-catenin signaling. Moreover, we developed an inhibitor that blocks the UBE2T-RACK1-Wnt/β-catenin axis and inhibits the GC progression. Together, our findings could offer new opportunities for Wnt/β-catenin signaling aberration GC patients.

## Results

### Aberrant Wnt/β-catenin signaling plays an important role in GC progression

**Fig. 1:**
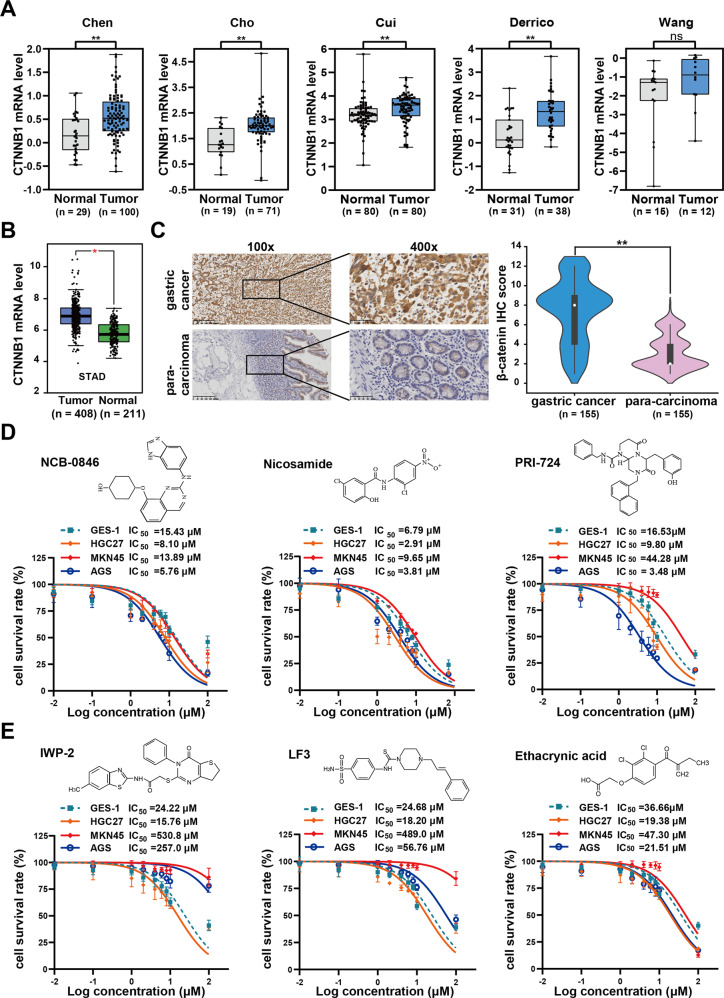
β-catenin expression level in GC and the effect of Wnt pathway inhibitors on GC cells. **A** Data mining for CTNNB1 (β-catenin) transcription level from the Oncomine database. **B** CTNNB1 (β-catenin) mRNA expression was analyzed by Gene Expression Profiling Interactive Analysis (GEPIA) database. **C** Immunohistochemistry (IHC) representative images of β-catenin and the relative statistical analysis of IHC scores. Data were expressed by the median (interquartile range, IQR). Paired *t* test was used to examine statistical significance (**P < 0.01). **D**, **E** Six kinds of current Wnt pathway inhibitors in a preclinical or clinical trial for other cancers have high cytotoxicity to GES-1 cells, **E** and three kinds of inhibitors cannot effectively suppress GC cells growth.

It is well-known that dysregulation of the Wnt/β-catenin signaling pathway is critically correlated with cancer initiation and progression [18]. In order to further examine the involvement of aberrant Wnt/β-catenin signaling pathway in GC, we performed data mining analysis in publicly available human GC datasets using the Oncomine platform. Data from five independent datasets consistently showed that the mRNA levels of β-catenin in GC tissues were significantly higher than those in normal counterparts (Fig. 1A). This was further confirmed by Gene Expression Profiling Interactive Analysis (GEPIA) (Fig. 1B). Immunohistochemistry (IHC) assay showed that β-catenin protein level was significantly upregulated (Fig. 1C), in consistency with the Wnt/β-catenin signaling pathway being activated in gastric cancer. Our present data thus strongly suggest that Wnt/β-catenin signaling hyperactivation is highly associated with GC progression.

### Current inhibitors targeting Wnt pathway components are either high cytotoxicity or low efficiency for GC

Next, we investigate whether previously developed Wnt pathway inhibitors in preclinical or clinical trials possess inhibitory effects on GC cell growth. We chose to test the effects of nine inhibitors in clinical or preclinical trials for other cancers on cell viability in HGC27, MKN45, and AGS cells, using GES-1 (Human gastric mucosal cells) as control. Our data showed that three kinds of the inhibitors (NCB-0846, nicosamide, and PRI-724) have obvious cytotoxicity to GES-1 (Fig. 1D), whereas the others (IWP-2, LF3, ethacrynic acid, ETC-159, LGK-974, and Wnt-C59) cannot effectively suppress HGC27, AGS, and MKN45 cells growth as well as have cytotoxicity to GES-1 (Fig. 1E and Supplementary Fig. S1). Our data thus strongly indicated that these inhibitors targeted Wnt/β-catenin signaling has either potential side effects on GES-1 or off-target effects on GC cells.

### Screening novel therapeutic targets for GC from the key components of β-catenin destruction complex

**Fig. 2:**
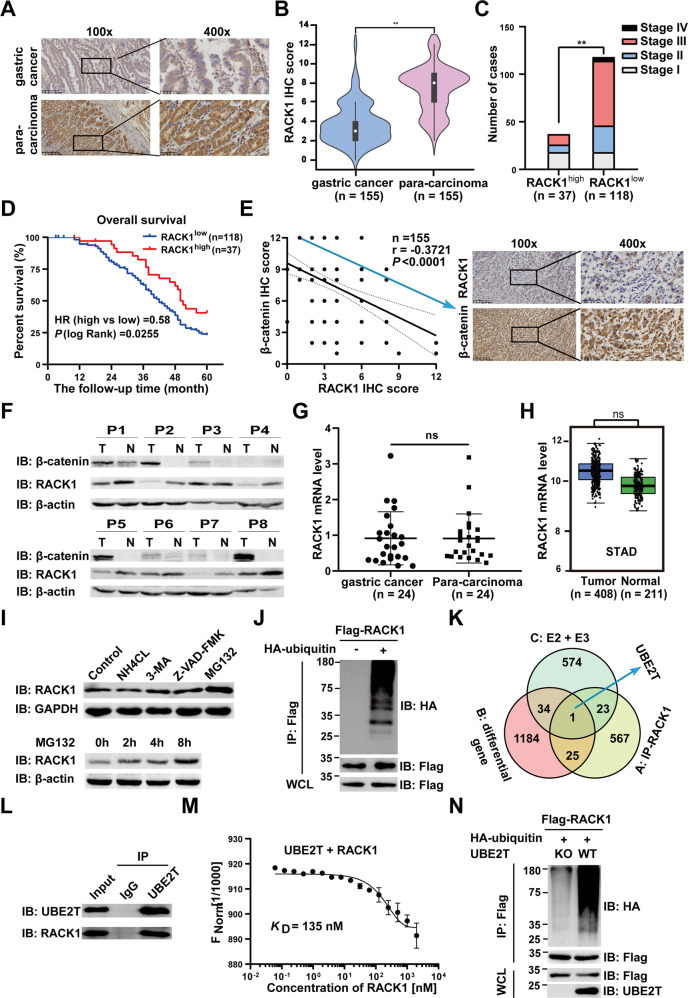
Clinical significance of RACK1 in gastric cancer and identification of its upstream regulator UBE2T. **A**, **B** The protein level of RACK1 detected by Immunohistochemistry (IHC), **A** representative image of RACK1 and **B** the relative statistical analysis of IHC scores. Data were expressed by the median (interquartile range, IQR). A paired *t* test was used to examine statistical significance (**P < 0.01). **C** The correlation between RACK1 protein level and the clinicopathological stage. Pearson chi-square test was used to examine statistical significance (**P < 0.01). **D** The Kaplan–Meier survival analysis of GC patients with RACK1^low^ (*n* = 118) or RACK1^high^ (*n* = 37) group. **E** Correlational analysis between RACK1 and β-catenin immunohistochemistry (IHC) score in GC tissues and representative images of IHC. Spearman correlation coefficient = −0.3721; *P* < 0.0001. *n* = 155. **F** Protein level of RACK1 and β-catenin in eight paired GC tissues and para-carcinoma tissues detected by western blotting. **G** Relative expression of RACK1 mRNA by qRT-PCR (*n* = 24). **H** RACK1 mRNA level by gene expression profiling interactive analysis (GEPIA). **I** The protein level of RACK1 after treated with 10 μM of the lysosome inhibitor NH4Cl and 3-MA, the caspase inhibitor Z-VAD-FMK, and the proteasome inhibitor MG132 for 8 h in HGC27 cells, and then cell lysates were analyzed by western blot with indicated antibodies. **J** Flag-tagged RACK1 and HA-tagged Ub plasmids were co-transfected into AGS cells for 36 h, followed by cell lysate preparation and IP assay with anti-Flag beads followed by immunoblotting with indicated antibodies. IP immunoprecipitates, WCL whole-cell lysates. **K** Venn diagram of genes from mass spectrometry analysis, mRNA microarray data analysis, and human E2s plus E3s. **L** The endogenous interaction of UBE2T and RACK1 was tested in AGS cells. **M** The binding of fluorescently labeled UBE2T to RACK1 is analyzed with microscale thermophoresis (MST) assay. RACK1 is titrated from 61 nM to 2 μM. The change in the thermophoretic signal leads to a *K*_D_ = 135 nM. **N** Wild or *UBE2T*^*−/−*^ AGS cells were transiently transfected with plasmids encoding Flag-tagged RACK1 and HA-tagged ubiquitin. Thirty hours after transfection, cells were treated with MG132 for 8 h (10 μM). Cell lysates were analyzed by immunoprecipitation with anti-Flag and western immunoblotting with indicated antibodies.

β-catenin is a crucial signaling transducer in Wnt/β-catenin signaling. The β-catenin protein destruction complex composed of adenomatous polyposis coli (APC), glycogen synthase kinase 3 (GSK3), and AXIN tightly controls the fate of β-catenin [19]. Therefore, to reveal the clinical relevance of β-catenin destruction complex key components (GSK3β, APC, RACK1, and AXIN) with β-catenin protein in GC, we then examined the expression of them in 155 cases of GC surgically resected specimens, which were divided into low-expression group and high-expression group. Our data showed that the protein levels of GSK3β, APC, and AXIN have no significant differences in 155 GC tissues compared with paired para-carcinoma tissues (Supplementary Fig. S2a), and have no significant correlation with the prognosis of these patients (Supplementary Fig. S2b, c). Interestingly, the protein level of RACK1 is decreased in 155 GC tissues compared with paired para-carcinoma tissues (Fig. 2A, B), and the level of RACK1 is negatively correlated with the depth of invasion, lymph node metastasis, clinical stage of 155 GC patients (Supplementary Table S1 and Fig. 2C). Moreover, survival analysis revealed that low RACK1 protein level correlates with reduced overall survival in those patients (Fig. 2D). To determine the effect of RACK1 on GC cell proliferation, we knock out the *RACK1* with CRISPR/Cas9 technology in HGC27, AGS, and MKN45 cells (Supplementary Fig. S3a), and colony-formation assay showed that the cloned numbers of *RACK1*^*−/−*^ cell was more than wild type (Supplementary Fig. S3b). Previous studies have shown that β-catenin protein level is negatively correlated with RACK1 in GC [17]. Our IHC data further confirmed that the level of β-catenin is negatively correlated with RACK1 in GC tissues (Fig. 2E), which was consistent with western blot findings using eight GC patients’ samples (Fig. 2F). Taken together, these findings indicated that RACK1 acts as a component from β-catenin destruction complex to play a vital role in GC progression, rather than GSK3β, APC, and AXIN.

### RACK1 as a key component of β-catenin destruction complex is modified by UPS in GC progression

In order to determine how RACK1 protein level was downregulated in GC tissues, we tested the mRNA expression of RACK1 in 24 paired gastric cancer and para-carcinoma tissues. We found that RACK1 expression level was similar between gastric cancer tissues and paired para-carcinoma tissues (Fig. 2G), which is consistent with GEPIA (Fig. 2H) and Oncomine database (Supplementary Fig. S4a). Therefore, we hypothesized that RACK1 was modified by cellular post-translational processing. Then, we investigated the effect of lysosomal inhibitor (NH4Cl, 3-MA), apoptosis inhibitor Z-VAD-FMK or proteasome inhibitor MG132 on the overall level of RACK1. As shown in Fig. 2I, only MG132 could increase the level of RACK1, indicating that RACK1 can be degraded by the ubiquitin–proteasome system (UPS). In agreement, we observed RACK1 ubiquitination in AGS (Fig. 2J) and HEK-293T cells (Supplementary Fig. S4b). To further gain insights into the underlying mechanisms that RACK1 is ubiquitinated and degraded, we searched for RACK1-interacting proteins in HGC27 cells. The interacting proteins were identified by affinity purification with an anti-FLAG antibody followed by liquid chromatography–tandem mass spectrometry analysis. Combined with mRNA microarray data from 16 GC patient’s samples and human-associated E2s plus E3s, we identified a ubiquitin-conjugating enzyme UBE2T (Fig. 2K and Supplementary Fig. S4c–e). Consistently, Co-IP assays showed that RACK1 indeed interacts with UBE2T (Fig. 2L and Supplementary Fig. S4f–h). Moreover, we also found the interaction of RACK1 with UBE2T in microscale thermophoresis (MST) assay, which indicated that RACK1 directly interacts with UBE2T (Fig. 2M). Previous studies showed that UBE2T is a conjugating enzyme (E2) which induces the ubiquitination and degradation of targeted proteins, such as BRCA1 and FANCD2 [20, 21]. Here, we investigated whether UBE2T could target RACK1 for ubiquitination. Our data showed that UBE2T promotes the ubiquitination of RACK1 (Fig. 2N and Supplementary Fig. S4i). To conclude, these results demonstrated that UBE2T leads to RACK1 protein homeostasis disorder via enhancing its ubiquitination.

### UBE2T induces the ubiquitination and degradation of RACK1 at K172, K225, and K257 residues independent of E3 ligase

**Fig. 3:**
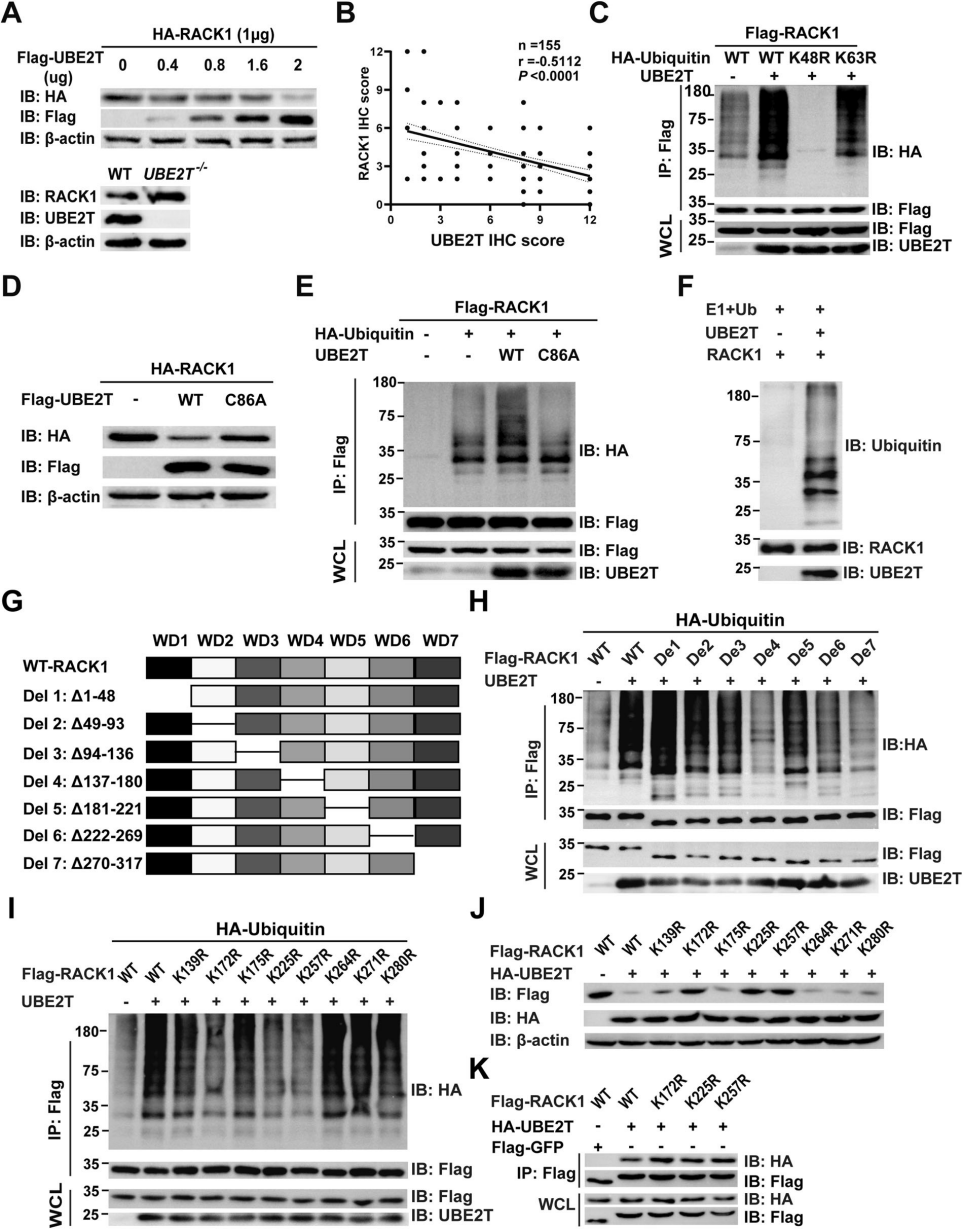
UBE2T induces ubiquitination and proteasomal degradation of RACK1 at K172, K225, and K257 independent of any E3 ligase. **A** HEK-293T cells were transiently transfected with plasmids encoding HA-tagged RACK1, along with the indicated amounts of a plasmid encoding Flag-tagged UBE2T 24 h after transfection, cell lysates were analyzed by western blot with indicated antibodies. Top: the RACK1 protein level in wild or *UBE2T*^−*/−*^ AGS cells was analyzed by western blot with indicated antibodies (bottom). **B** Correlational analysis between UBE2T and RACK1 immunohistochemistry (IHC) score in GC tissues. Spearman correlation coefficient = −0.5112; *P* < 0.0001. *n* = 155. **C** HEK-293T cells were transiently transfected with plasmids encoding Flag-tagged RACK1 and UBE2T, along with plasmids encoding HA-tagged wild ubiquitin or indicated mutant ubiquitin. Sixteen hours after transfection, cells were treated with MG132 for 8 h (10 μM). Cell lysates were analyzed by immunoprecipitation with anti-Flag and western immunoblotting with indicated antibodies. **D** HEK-293T cells were transiently transfected with plasmids encoding HA-tagged RACK1, along with a plasmid encoding Flag-tagged wild-type UBE2T or UBE2T^C86A^ mutant. C86A: the 86th cysteine was substituted to alanine. Twenty-four hours after transfection, cell lysates were analyzed by western blot with indicated antibodies. **E** HEK-293T cells were transiently transfected with plasmids expressing Flag-tagged RACK1 and HA-tagged ubiquitin, along with plasmids encoding wild UBE2T or UBE2T^C86A^ mutant. Sixteen hours after transfection, cells were treated with MG132 for 8 h (10 μM). Cell lysates were analyzed by immunoprecipitation with anti-Flag and western immunoblotting with indicated antibodies. **F** Recombinant RACK1 proteins were subjected to in vitro ubiquitination assay in the absence or presence of in vitro-translated wild-type UBE2T, and western immunoblotting with indicated antibodies. **G** The schematic diagram of RACK1 domain. **H** HEK-293T cells were transiently transfected with plasmids expressing Flag-tagged the deletion of the indicated domain of RACK1 and HA-tagged ubiquitin, along with plasmid expressing UBE2T. **I** HEK-293T cells were transiently transfected with plasmids expressing Flag-tagged of indicated RACK1 mutant plasmids and HA-tagged ubiquitin plasmids, along with plasmid expressing UBE2T. **J** Wild-type and lysine residual mutated Flag-tagged RACK1 plasmids were individually transfected into HEK-293T cells, with or without HA-tagged UBE2T. Cell lysates were analyzed by western blot with indicated antibodies. **K** A plasmid expressing Flag-tagged wild type, indicated mutant RACK1 or GFP were transfected into HEK-293T cells with a plasmid expressing HA-tagged UBE2T. Sixteen hours after transfection, cells were treated with MG132 for 8 h (10 μM). Cell lysates were analyzed by immunoprecipitation with anti-Flag and western immunoblotting with indicated antibodies.

To further explore the mechanism that UBE2T ubiquitinated RACK1, we investigated whether UBE2T can degrade RACK1. Our data showed that the overexpression of UBE2T leads to a significant dose-dependent reduction in total protein levels of RACK1 and *UBE2T*^*−/−*^ AGS cells had higher RACK1 protein levels than wild AGS cells (Fig. 3A). From a clinical point of view, IHC assay further showed that the level of UBE2T is negatively correlated with RACK1 in GC tissues (Fig. 3B and Supplementary Fig. S5a). Furthermore, our data confirmed that UBE2T promotes the K48 linked, but not K-63 linked polyubiquitination of RACK1 (Fig. 3C). Next, we sought to understand how UBE2T functions in GC progression and whether it is mediated through the promotion of the ubiquitination and degradation of RACK1. Our data showed that the overexpression of UBE2T could induce RACK1 degradation, but the catalytic mutant UBE2T^C86A^ failed to do so (Fig. 3D). Meanwhile, UBE2T overexpression promotes polyubiquitination of RACK1, which depended on the catalytic site cysteine 86 (Fig. 3E). Furthermore, the interaction of UBE2T with RACK1 was not dependent on the cysteine 86 (Supplementary Fig. S5b). Previous studies showed that RACK1 may function as a substrate or an E3 ligase itself [22, 23]. As shown in Fig. 3F, in vitro ubiquitination assay is consistent with the hypothesis that UBE2T can induce the ubiquitination and degradation of RACK1 without any E3 ligase.

We next sought to investigate the ubiquitination sites of RACK1 essential for UBE2T-mediated RACK1 ubiquitination. Bioinformatics analysis showed that RACK1 contains seven function domains (Fig. 3G). Then we tested the effect of these domains separately on the ubiquitination of RACK1 by UBE2T. The results showed that WD4, 6, and 7 but not WD 1, 2, 3, or 5 were essential for ubiquitination of RACK1 (Fig. 3H). We found that there are three lysines in WD4 (K139, K172, and K175), three lysines in WD6 (K225, K257, and K264), and two lysines in WD7 (K271 and K280). In order to identify the specific ubiquitination sites of RACK1, we generated a series of lysine (K) to arginine (R) mutants of RACK1 (K139R, K172R, K175R, K225R, K257R, K264R, K271R, and K280R) based on the above experimental data. Our data showed that UBE2T-induced ubiquitination of RACK1-K172R, K225R, and K257R was significantly abolished, suggesting that the K172, K225, and K257 residues of RACK1 are the major ubiquitination sites (Fig. 3I). Furthermore, we investigated whether these mutations affect the degradation of RACK1 by UBE2T or the interaction of RACK1 with UBE2T. We found that mutations of K172R, K225R, and K257R abolished the degradation of RACK1 induced by UBE2T but not the interaction between RACK1 and UBE2T (Fig. 3J, K). These data indicated that UBE2T promotes the degradation of RACK1 by inducing its polyubiquitination at K172, K225, and K257 without any E3 ligase.

### UBE2T promotes β-catenin translocation into the nucleus and hyperactivates Wnt/β-catenin signaling pathway in GC by inducing RACK1 degradation

**Fig. 4:**
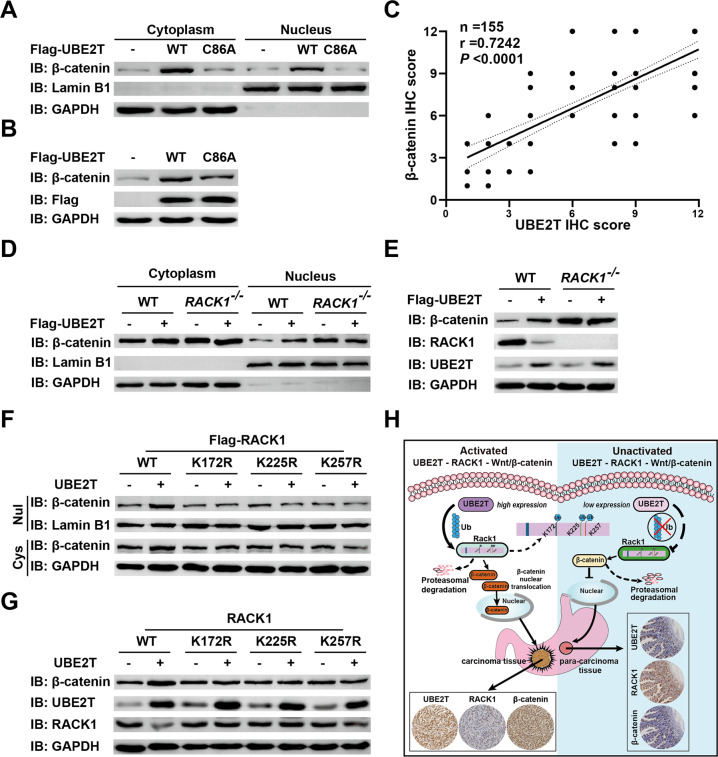
The UBE2T-RACK1 axis promotes the hyperactivation of the Wnt/β-catenin signaling pathway. **A**, **B** The wild-type UBE2T plasmids or UBE2T^C86A^ mutant plasmids were transfected into HEK-293T cells. Twenty-four hours after transfection, total cell lysates (**B**), cytoplasmic, or nuclear lysates (**A**) were analyzed by western blot with the indicated antibodies. **C** Correlational analysis between UBE2T and β-catenin immunohistochemistry (IHC) score in GC tissues. Spearman correlation coefficient = 0.7242; *P* < 0.0001. *n* = 155. **D**, **E** Mock or UBE2T plasmids were individually transfected into in wild-type or RACK1-deficient HEK-293T cells. Twenty-four hours after transfection, total cell lysates (**E**), cytoplasmic, or nuclear lysates (**D**) were analyzed by western blot with the indicated antibodies. **F**, **G** Wild-type and indicated lysine residual mutated RACK1 plasmids were individually transfected into HEK-293T cells with or without UBE2T. Twenty-four hours after transfection, total cell lysates (**G**), cytoplasmic or nuclear lysates (**F**) were analyzed by western blot with the indicated antibodies. **H** Schematic diagram of the mechanism that UBE2T drives gastric carcinogenesis.

Previous studies have shown that RACK1 acts as a negative regulator of the Wnt/β-catenin signaling pathway by stabilizing the β-catenin destruction complex [17]. Given that UBE2T significantly mediated the ubiquitination and degradation of RACK1, we were particularly interested in exploring whether UBE2T could promote Wnt/β-catenin signaling pathway hyperactivation by inducing ubiquitination and degradation of RACK1. First, we investigated whether UBE2T was involved in the hyperactivation of the Wnt/β-catenin signaling pathway. Our data showed that overexpression of UBE2T markedly increased the amount of cytoplasmic and nuclear β-catenin, which depended on its catalytic site cysteine 86 (Fig. 4A, B). IHC assay showed that the UBE2T level is positively correlated with β-catenin in GC tissues (Fig. 4C and Supplementary Fig. S5c). Then, in order to verify whether the above-mentioned functions of UBE2T was dependent on RACK1, we generated RACK1 knockout cells to test the hyperactivation of the Wnt/β-catenin signaling pathway by UBE2T. The data showed that overexpression of UBE2T did not induce a significant increase in the amount of cytoplasmic and nuclear β-catenin in RACK1 knockout cells (Fig. 4D, E). Meanwhile, we also found that co-expression of UBE2T with wild-type RACK1 but not with the lysine mutants at the ubiquitination sites increased the amount of cytoplasmic and nuclear β-catenin (Fig. 4F, G). All of the data above demonstrated that UBE2T hyperactivates the Wnt/β-catenin signaling pathway by inducing polyubiquitination of RACK1 at K172, K225, and K257 and its degradation, thereby promoting GC progression (Fig. 4H).

### UBE2T contributes to GC progression

**Fig. 5:**
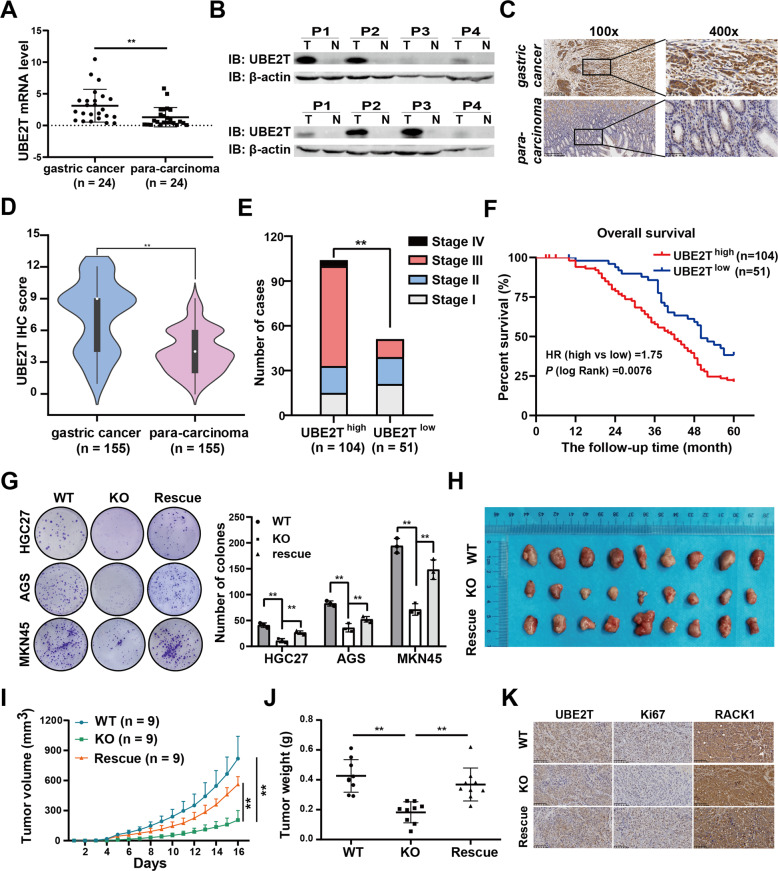
UBE2T expression is increased and negatively correlates with GC progression. **A** The UBE2T mRNA level detected by qRT-PCR in 24 paired gastric cancer tissues and corresponding para-carcinoma tissues. **B** The Relative expression of UBE2T in eight paired gastric cancer tissues and para-carcinoma tissues detected by western blotting. **C**, **D** The protein level of UBE2T detected by immunohistochemistry (IHC). **C** Representative images of UBE2T, and **D** the relative statistical analysis of IHC scores. Data were expressed by the median (interquartile range, IQR). A paired *t* test was used to examine statistical significance (**P < 0.01). **E** The correlation between UBE2T expression and the clinicopathological stage. Pearson chi-square test was used to examine statistical significance (**P < 0.01). **F** The Kaplan–Meier survival analysis of patients with UBE2T^low^ (*n* = 51) or UBE2T^high^ (*n* = 104) group. **G** Colony-formation assay in wild type, *UBE2T*^−*/*−^, or *UBE2T*^*−/−*^ complemented with *UBE2T* HGC27, AGS, and MKN45 cells. Student’s *t* test was used to examine statistical significance (mean ± S.D., *n* = 3, **P < 0.01, *P < 0.05). **H**–**K** Wild type, *UBE2T*^*−/−*^, or *UBE2T*^*−/*−^ complemented with *UBE2T* MKN45 cells were intratumor injected in nude mice, respectively. **H** Shown are volumes, **I** weight, and **J** representative image of the intratumor. **K** The representative immunohistochemical images of UBE2T, RACK1, and Ki-67 in intratumor tumors of BALB/C mouse. Scale bar, 40 μm. Student’s *t* test was used to examine statistical significance (mean ± S.D., *n* = 9, **P < 0.01, *P < 0.05). WT wild type, KO *UBE2T*^−*/*−^, rescue *UBE2T*^*−/*−^ complemented with *UBE2T*.

Interestingly, our previous study showed that UBE2T plays a vital role in GC progression [24]. To confirm the effects of UBE2T on GC, we further validated the previous data by more GC samples and rescue assay in different GC cells. Data from Oncomine and GEPIA datasets showed that the mRNA level of UBE2T was significantly higher in GC tissues (Supplementary Fig. S6a, b), Moreover, GEPIA database showed that the mRNA level of UBE2T was also upregulated in 24 additional cancer types (Supplementary Fig. S7). We studied the mRNA and protein levels of UBE2T in GC patients' samples: 24 samples for qRT-PCR assay, 8 for western blotting assay, and 155 for IHC assay (Fig. 5A–D). We found that mRNA and protein levels of UBE2T are obviously increased and UBE2T expression is positive correlated with tumor size, depth of invasion, lymph node metastasis, clinical stage (Supplementary Table S2 and Fig. 5E), and poor prognosis (Fig. 5F). We found that the protein level of UBE2T is upregulated in AGS, HGC27, and MKN45 cell lines (Supplementary Fig. S8a). We knock out the UBE2T with CRISPR/Cas9 technology and rescued it in HGC27, AGS, and MKN45 cells (Supplementary Fig. S8b). Colony-formation assay showed that the cloned numbers of *UBE2T*^−*/−*^ cell was less than wild type or *UBE2T*^−*/−*^ complemented with *UBE2T* (Fig. 5G). We further verified the effect of UBE2T on tumor growth in vivo. We found that the growth of *UBE2T*^−*/−*^ MKN45 xenograft was slower and smaller than wild type or *UBE2T*^−*/−*^ complemented with *UBE2T*, and Ki-67 level is lower in *UBE2T*^*−/*−^, whereas RACK1 level is higher in *UBE2T*^−*/*−^ (Fig. 5H–K), suggesting that UBE2T knockout suppresses malignant progression in vivo and UBE2T is a promising target for GC therapy.

### Identification of UBE2T inhibitors based on computational virtual screens and biological effectiveness assay

**Fig. 6:**
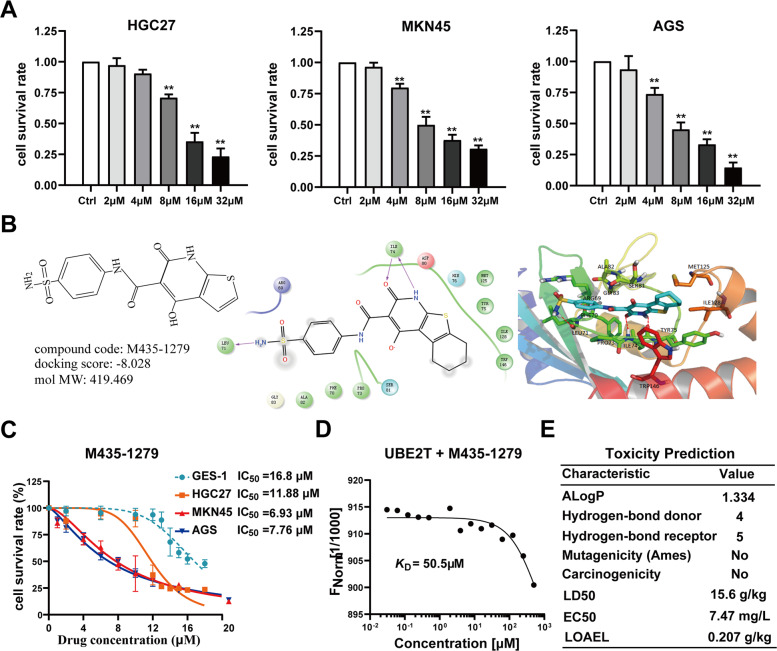
Identification of UBE2T inhibitor. **A** Cell survival rate of HGC27, AGS, and MKN45 cells after 48 h treated with M435-1279. Cell viability was detected by 3-(4,5-dimethyl-2-thiazolyl)-2,5-diphenyl-2-H-tetrazolium bromide (MTT) assay. One-way analysis of variance (ANOVA) was used to examine statistical significance (mean ± S.D., *n* = 6, **P < 0.01, *P < 0.05). **B** A simulation snapshot of M435-1279 with the allosteric site of UBE2T by molecular dynamics simulations. Sticks defined as the compounds and the active sites interacted with UBE2T, red thread dotted lines defined as hydrogen bonds between the compounds and UBE2T. For the compound, hydrogen: white, carbon: blue, oxygen: red, nitrogen: dark blue, and sulfur: yellow. **C** The effect of M435-1279 on the growth of HGC27, AGS, MKN45, and GES-1 (as control), respectively. Cell viability was detected by 3-(4,5-dimethyl-2-thiazolyl)-2,5-diphenyl-2-H-tetrazolium bromide (MTT) assay. **D** The binding of fluorescently labeled UBE2T to M435-1279 is analyzed with microscale thermophoresis (MST) assay. The M435-1279 is titrated from 31 nM to 500 μM. The change in the thermophoretic signal leads to a Kd = 50.5 μM. **E** The toxicity of M435-1279 is predicted based on TOPKAT analysis. ALogP lipophilicity (<5 value shows good lipophilicity), LD50 50% lethal dose of a chemical that kills 50% of a sample population, EC50 water flea EC50, 50% effective concentration, LOAEL lowest-observed-adverse-effect level.

The above studies strongly suggested that UBE2T plays an important role in promoting the progression of GC owing to UBE2T-induced ubiquitination and degradation of RACK1. We attempted to identify novel small-molecule inhibitors for UBE2T by performing computational virtual screens. The docking results showed 115 hits from the ChemDiv and SPECS small-molecular compounds library. According to the docking scores, 18 compounds were selected as potential UBE2T-binding ligands for further screening (Supplementary Fig. S9). First, we tested the effects of these 18 inhibitors above on the growth of HGC27, AGS, and MKN45 cells by an MTT assay. The data showed that only M435-1279 significantly inhibited the growth of HGC27, AGS, and MKN45 cells (Fig. 6A and Supplementary Fig. S10), and AG-690/12244866 significantly inhibited HGC27 but not AGS and MKN45 cells (Supplementary Fig. S11a, b). Computational virtual analyses predicted that M435-1279 inserts themselves into the catalytic pocket of UBE2T and has a higher docking score than AG-690/12244866 (Fig. 6B and Supplementary Fig. S11c, d). Then, we tested the cytotoxic effect of M435-1279 and AG-690/12244866, the data showed that M435-1279 (Fig. 6C) has lower cytotoxicity to GES-1 than AG-690/12244866 (Supplementary Fig. S11b), which indicated that the M435-1279 could be used as a more potent drug for GC therapy. We used the MST assay to determine the binding affinity of M435-1279 to UBE2T protein. The results suggested a K*D* value of 50.5 μM for M435-1279 binding to UBE2T (Fig. 6D). The results of toxicity prediction were shown in Fig. 6E, the M435-1279 has low toxicity including no carcinogenicity, mutagenicity, and high LD50, LOAEL, and low environmental toxicity (water flea EC50), which is consistent with our data that LD50 > 5 g/kg (actually nontoxic).

### UBE2T inhibitor M435-1279 blocks β-catenin translocation into nuclear and GC progression

**Fig. 7:**
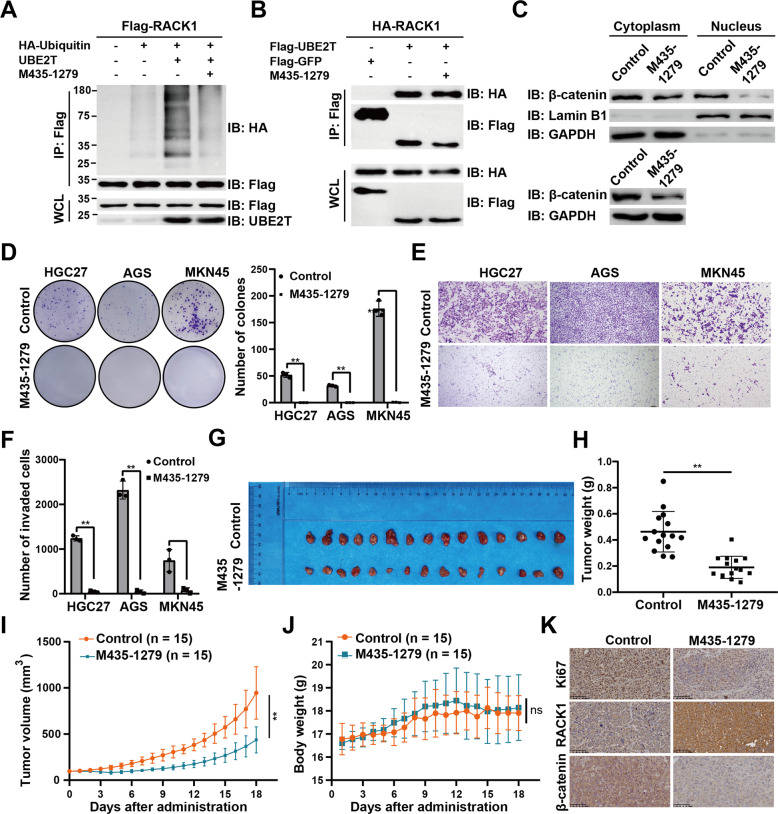
Antagonism of the Wnt pathway by UBE2T inhibitor M435-1279 in GC. **A** The effect of M435-1279 on ubiquitination of RACK1. Lysates from 293T cells expressing the indicated plasmids after 48 h treated with M435-1279 (11.88 μM) were immunoprecipitated (IP) with an anti-Flag followed by immunoblotting against indicated antibodies. **B** The effect of M435-1279 on the interaction of UBE2T with RACK1. M435-1279 did not affect UBE2T-Rack1 interaction by immunoprecipitated assays. Lysates from 293T cells expressing the indicated plasmids after 48 h treated with M435-1279 (11.88 μM) were immunoprecipitated (IP) with an anti-Flag followed by immunoblotting with indicated antibodies. **C** The effect of M435-1279 on the total or nuclear β-catenin expression of HGC27 cells. Forty-eight hours after M435-1279 treatment, total cell lysates (bottom), cytoplasmic, or nuclear lysates (top) were analyzed by western blot with the indicated antibodies (Lamin B1 as a nuclear loading control, GAPDH as a cytoplasmic loading control). **D**–**F** Effect of M435-1279 treatment on tumor (**D**) proliferation and (**E**, **F**) migration in HGC27, AGS, and MKN45 cells. Proliferation and migration ability were detected by colony-formation assay and Transwell assay, respectively. Student’s *t* test was used to examine statistical significance (mean ± S.D., *n* = 3, **P < 0.01, *P < 0.05). **G**–**K** MKN45 cells were intratumor injected in nude mice. The compound was dosed by intratumor injection at a single dose of 5 mg/kg/day, and DMSO was used as a control group. Shown are (**G**) representative image, (**H**) tumor weights, (**I**) tumor volumes, and (**J**) body weights. **K** The representative immunohistochemical images of Ki-67, RACK1, and β-catenin in intratumor tumors of mouses. Scale bar, 40 μm. Student’s *t* test was used to examine statistical significance (mean ± S.D., *n* = 15, **P < 0.01, *P < 0.05).

The data above showed that UBE2T contributes to GC progression mainly through inducing ubiquitination and degradation of RACK1 to hyperactivate the Wnt/β-catenin signaling pathway. Therefore, we hypothesized that the binding of M435-1279 to UBE2T may block UBE2T-RACK1 axis-induced hyperactivation of the Wnt/β-catenin signaling pathway. We examined the effects of the compound on the interaction of RACK1 with UBE2T, the ubiquitination of RACK1 by UBE2T, and the hyperactivation of the Wnt/β-catenin signaling pathway induced by UBE2T. The data showed that the compound didn’t block the interaction (Fig. 7B) between RACK1 and UBE2T but obviously inhibited the ubiquitination of RACK1 (Fig. 7A) and the hyperactivation of the Wnt/β-catenin pathway (Fig. 7C).

Next, we investigated whether the compound could inhibit the UBE2T-derived GC progression. The colony-formation and Transwell assay showed that the compound could suppress the proliferation and migration of HGC27, AGS, and MKN45 cells (Fig. 7D–F). Then, we did pilot experiments to identify an optimal concentration of 5 mg/kg/day for the following mouse experiment (Supplementary Fig. S11e). In the CDX model, we observed that the tumor growth was much slower in the M435-1279 treatment group than the control group, whereas body weights were similar (Fig. 7G–J). Furthermore, IHC assay showed that the expression of Ki-67 and β-catenin proteins were lower and RACK1 proteins were higher in the M435-1279 treatment group (Fig. 7K).

Taken together, these data demonstrated that M435-1279 shows a potent in vitro and in vivo efficacy in inhibiting the growth of GC, and could be a promising starting point for the development of UBE2T inhibitor therapeutics.

## Discussion

GC appears to be heterogeneous since both genomic alterations and environmental factors were universally involved in gastric carcinogenesis, which impedes the development of targeted drugs [25, 26]. Trastuzumab is the FDA-approved targeted drug for GC [27, 28]; however, the prolonged survival time of unresectable advanced GC patients treated with chemotherapy plus trastuzumab is ~2.7 months [4]. Previous studies showed that Wnt/β-catenin signaling hyperactivation could be triggered by virulence factor CagA and VacA of *Helicobacter pylori* infection in gastric carcinogenesis [29–32]. Our study also demonstrated that dysregulation of the Wnt/β-catenin signaling pathway is implicated in GC progression, which consists of previous findings that up to 30% of GC patients are Wnt/β-catenin signaling hyperactivation associated [33, 34]. Therefore, it is necessary to develop new therapeutic approaches targeting this pathway for GC patients with Wnt/β-catenin signaling hyperactivation.

Currently, inhibitors targeting the Wnt/β-catenin signaling pathway tend to be promising drugs for anti-tumor therapy [25]. It is well-known that these inhibitors mainly target the enzyme porcupine, WNT ligands and their receptors, frizzled receptors, the β-catenin-CBP complex, and so on [35, 36]. However, our data demonstrated current inhibitors in a preclinical or clinical trial for other cancers are either high cytotoxicity or low efficiency for GC, implying that these potential inhibitors targets are not appropriate for GC therapy due to their indispensable physiological functions or GC heterogeneity. Thus, it is essential to screen more valuable inhibitor targets from other components of the Wnt/β-catenin signaling pathway. Although the previous study has shown that the destabilization of the β-Catenin destruction complex plays a vital role in GC progression, no effective inhibitor targeting these complex components has been yet reported in GC [18]. Our data revealed that the decreased level of scaffolding protein RACK1 from β-catenin destruction complex components is significantly associated with poor prognosis of GC patients, whereas other scaffolding proteins are irrelevant to patient prognosis. Therefore, it may be possible to identify a cancer-specific target from the upstream of RACK1.

Our data showed that RACK1 is degraded by the ubiquitin–proteasome system rather than autophagy–lysosome pathway or apoptotic pathway in GC. Then, we identified UBE2T as a crucial regulator of RACK1 via mass spectrometry analysis combined with Affymetrix GeneChip^®^arrays analysis, and found that UBE2T strongly promotes the K48-linked polyubiquitination and degradation of RACK1 depends on its catalytic site cysteine 86 in HEK-293T cells [20]. We further confirmed their clinical-negative relevance between UBE2T and RACK1 in GC patients’ tissue specimens. Meanwhile, our data demonstrated that UBE2T could catalyze the ubiquitination of RACK1 independent of any E3 ligases in-tube ubiquitination assay. It is well-known that lysine selection is essential for the generation of diverse substrate–Ub structures, targeting proteins to different fates [37]. However, the specific ubiquitination sites of RACK1 remain unclear so far [22]. Our studies found that the lysine K172, K225, and K257 residues of RACK1 are responsible for UBE2T-mediated RACK1 ubiquitination and degradation. Our data further showed that UBE2T promotes the translocation of β-catenin into nuclear dependent on these three lysine residues of RACK1. Taken together, UBE2T-mediated RACK1 degradation drives GC progression through the nuclear accumulation of β-catenin and Wnt/β-catenin signaling hyperactivation, suggesting that RACK1 upstream regulator UBE2T functions as a potential target for GC therapy.

Previous studies have shown that UBE2T, also known as HSPC150, acts as a member of the E2 family of the ubiquitin–proteasome pathway [38–40]. Several studies demonstrated that UBE2T expression is remarkably upregulated in several cancers [41–43], which is consistent with expression data from GEPIA database [44]. Our data further confirmed that the overexpression of UBE2T is associated with poor prognosis of GC patients, and UBE2T knockout significantly inhibits GC progression. Thus, UBE2T may act as an important oncogene that triggers GC progression. Consistent with the above-mentioned mechanism findings that UBE2T promotes GC progression via Wnt/β-catenin signaling activation, it is attractive to develop inhibitors targeting cancer-specific Wnt/β-catenin signaling regulator UBE2T for GC therapy.

Despite the biological functions of UBE2T have ever been well studied in breast cancer and Fanconi anemia [20, 21], none therapeutic inhibitors targeting UBE2T have been reported yet [45, 46]. Here, we identified a novel UBE2T inhibitor M435-1279 through screening Chemdiv and SPECS small-molecular compounds library based on its catalytic site cysteine 86. Our data showed that the M435-1279 binds to UBE2T, and significantly suppresses the process of RACK1 ubiquitination and β-catenin translocation into nuclear. Besides, our data further validated that M435-1279 remarkably inhibits tumor growth of GC in vivo and vitro. Interestingly, we found that M435-1279 has low cytotoxicity to normal cells of gastric mucosa at effective inhibition concentration for GC cells, suggesting that M435-1279 could suppress GC progression through UBE2T-medicated Wnt/β-catenin hyperactivation without impairing its physiological function in the meantime. Therefore, UBE2T is a valuable inhibitor target regulating the Wnt/β-catenin signaling pathway in GC, and its specific inhibitor M435-1279 could be a potentially effective leading compound for GC therapy.

In this study, we found that current Wnt pathway inhibitors being studied in preclinical or clinical settings for other cancers are either too cytotoxic or insufficiently efficacious as effective GC therapeutics. Thus, we identified a novel target UBE2T which triggers GC progression by hyperactivating the Wnt/β-catenin signaling pathway through the ubiquitination and degradation of RACK1 at the lysine K172, K225, and K257 residues. Furthermore, we confirmed that a new UBE2T inhibitor M435-1279 could effectively suppress GC progression with lower cytotoxicity by blocking the hyperactivation of the Wnt/β-catenin signaling pathway in the meantime. Finally, we found that increased UBE2T level promotes GC progression via the ubiquitination of RACK1 independent of E3 ligase and identify a potent inhibitor providing a balance between growth inhibition and cytotoxicity as well, which offer a new opportunity for specific GC patients with aberrant Wnt/β-catenin signaling.

## Materials and methods

### Clinical samples

A total of 155 paired gastric cancer and para-carcinoma tissue samples were acquired with signed informed consent from Lanzhou University Second Hospital. All procedures involving human samples were approved by the Ethics Committee of Lanzhou University Second Hospital.

### Cell lines and culturing

Cultured GES-1 human gastric mucosal cells, gastric cancer cell lines, and 293T cells were purchased from the cell bank of the Chinese Academy of Sciences. All cell lines were cultured in Dulbecco’s Modified Eagle Medium (DMEM) supplemented with 10% fetal bovine serum, penicillin (100 mg/ml), and streptomycin (100 mg/ml) and incubated at 37° C in a humidified chamber with 5% CO_2_. All cells were passaged with 0.25% trypsin/2.21 mM EDTA in PBS when they achieved the confluency of 75–80%.

### Nuclear protein extraction

Nuclear Protein Extraction kit (Solarbio, R0050, China) was used to segregate the cytoplasm and nuclear protein. The Lamin B1 and GAPDH as the references of nuclear and cytoplasm protein, respectively.

### Immunohistochemistry

Tissue of patients and mice specimens were formalin-fixed and paraffin-embedded, and the process of immunohistochemistry was conducted referring to the article of Deng et al. [17]. All clinical samples were collected with informed consent. Generally, the score of specimens was evaluated by the intensity of the staining (0: no staining; 1: weak staining, 2: moderate staining, 3: strong staining) and the percentage of stained cells (0: 0%, 1: 1–24%, 2: 25–49%, 3: 50–74%, 4: 75–100%). The final immunohistochemical score is the product of the positive staining rate and the positive staining area score. We consider a 0–4 was low expression, and 6–12 was high expression.

### Cell viability assay

Approximately 4000 cells/well were seeded in a 96-well plate and treated with 11.8 μM M435-1279 for 48 h. Cell viability was measured OD_490_ after reaction 1 h with CellTiter 96^®^ AQ_ueous_ One Solution Reagent (G3582, Promega, USA).

### Animal studies

The BALB/C nude mice were purchased from the Beijing Charles River company. In total, 4 × 10^6^ MKN45 cells were suspended in 100 μl of PBS and 100 μl of Matrigel for intratumor injection. For small-molecule inhibitor, when the tumor size reached 75–100 mm^3^, mice were subjected to intratumor injections of 0.9% NaCl with DMSO, 5 mg/kg/day M435-1279 dissolve in 0.9% NaCl, respectively. All procedures involving mice and experimental protocols were approved by the Ethics Committee.

### In vivo ubiquitination assay

Cells expressing HA-Ubiquitin, no-tag-UBE2T, and flag-RACK1 were lysed by lysis buffer and then incubated with Flag-gel beads at 4 °C for 2 h. For ubiquitination assay in denaturing condition, cells were lysed by SDS lysis buffer [10 mM Tris-HCl (pH 7.5), 150 mM NaCl] containing 2% SDS for 5 min at 95 °C. Then, the lysates were diluted fourfold with dilution buffer [10 mM Tris-HCl (pH 7.5), 150 mM NaCl, 1% Triton X-100] [47], and then incubated with Flag-gel beads at 4 °C for 2 h. After washing, elution, neutralization, and boiling, the samples were assayed by western blot.

### In vitro ubiquitination assay

First, add reaction buffer (SK-10, Boston Biochem, USA) (10×) to solution contain UBE1 (E-305, Boston Biochem, USA), UBE2T (ab206015, Abcam, UK), RACK1 (TP305092, ORIGENE, USA) and Ubiquitin (U-100H, Boston Biochem, USA). Add a solution of Mg-ATP, and then bring the volume to 20 μl with ddH_2_O. Second, incubate the reaction at 37 °C for 2 h, add stop buffer and SDS-buffer. Finally, boil it for 5 min and perform a western blot assay.

### Statistical analysis

SPSS version 26.0 (IBM, Armonk, NY, USA) and GraphPad Prism 8.0 (GraphPad, San Diego, CA, USA) were used to perform statistical analyses and graphing, respectively. Pearson chi-square tests, independent sample *T* test, or paired *t* test were used to compare the difference between groups for categorical and continuous data, respectively. And the data were expressed as percentages, mean ± standard or median (interquartile range, IQR). *P* < 0.05 was considered significant.

### Primer and antibody

Primer sequence and antibody information are showed in Supplementary Tables S3, S4, and S5, respectively.

## Supplementary information

Fig. S1

Fig. S2

Fig. S3

Fig. S4

Fig. S5

Fig. S6

Fig. S7

Fig. S8

Fig. S9

Fig. S10

Fig. S11

Table S1

Table S2

Table S3

Table S4

Table S5

Supplementary Materials and Methods
