# Supplementary material for: A novel UBE2T inhibitor suppresses Wnt/β-catenin signaling hyperactivation and gastric cancer progression by blocking RACK1 ubiquitination
Source: Oncogene. 2020 Dec 15;40(5):1027–42. doi: 10.1038/s41388-020-01572-w (PMC7862066; doi:10.1038/s41388-020-01572-w)
Supplement: Supplementary file 1 — Fig. S1 [file 41388_2020_1572_MOESM1_ESM.pdf]

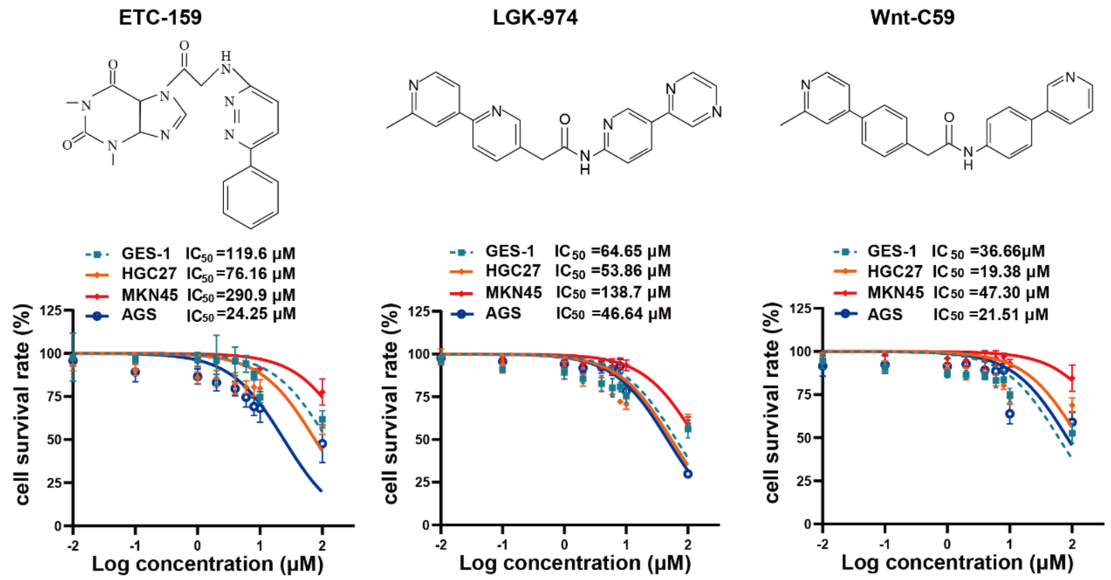


**Fig. S1** The effect of three Wnt pathway inhibitors (ETC-159, LGK-974 and Wnt-C59) on Cell viability of HGC27, AGS and MKN45 cells was detected by 3-(4,5-dimethyl-2-thiazolyl)-2,5- diphenyl-2-H-tetrazolium bromide (MTT) assay.
