# Supplementary material for: A novel UBE2T inhibitor suppresses Wnt/β-catenin signaling hyperactivation and gastric cancer progression by blocking RACK1 ubiquitination
Source: Oncogene. 2020 Dec 15;40(5):1027–42. doi: 10.1038/s41388-020-01572-w (PMC7862066; doi:10.1038/s41388-020-01572-w)
Supplement: Supplementary file 2 — Fig. S2 [file 41388_2020_1572_MOESM2_ESM.pdf]

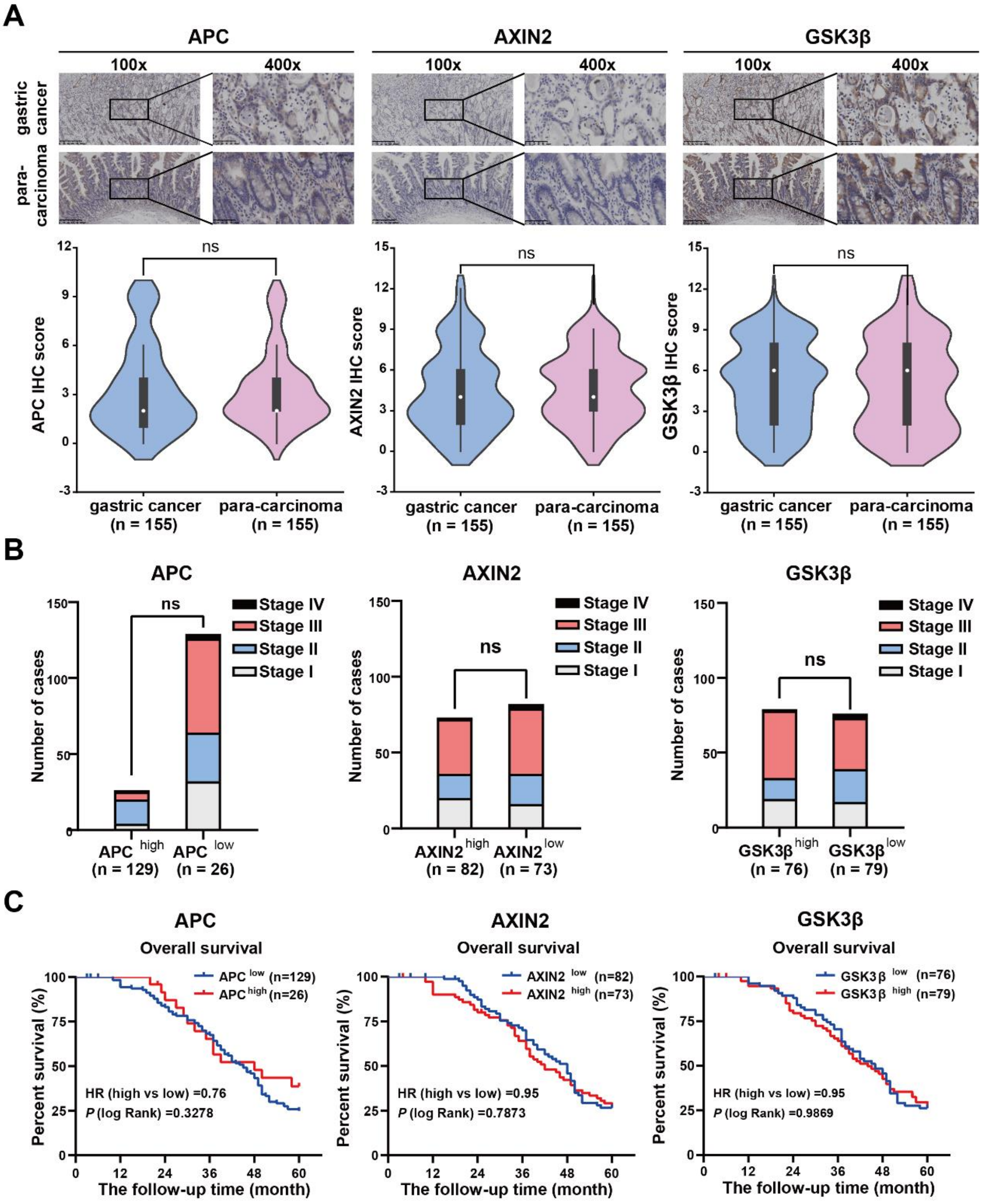

Fig. S2 a Representative image of IHC staining and the relative IHC scores (n=155) of AXIN, APC, and Gsk3β in GC tissues. Scale bar, 50 μm.(n=155). **b** Relationship between expression of AXIN, APC, Gsk3β and Clinical stage in Gastric Cancer. **c** Kaplan–Meier analysis of overall survival probability of AXIN, APC, and Gsk3β levels in GC (n=155).
