# Supplementary material for: A novel UBE2T inhibitor suppresses Wnt/β-catenin signaling hyperactivation and gastric cancer progression by blocking RACK1 ubiquitination
Source: Oncogene. 2020 Dec 15;40(5):1027–42. doi: 10.1038/s41388-020-01572-w (PMC7862066; doi:10.1038/s41388-020-01572-w)
Supplement: Supplementary file 3 — Fig. S3 [file 41388_2020_1572_MOESM3_ESM.pdf]

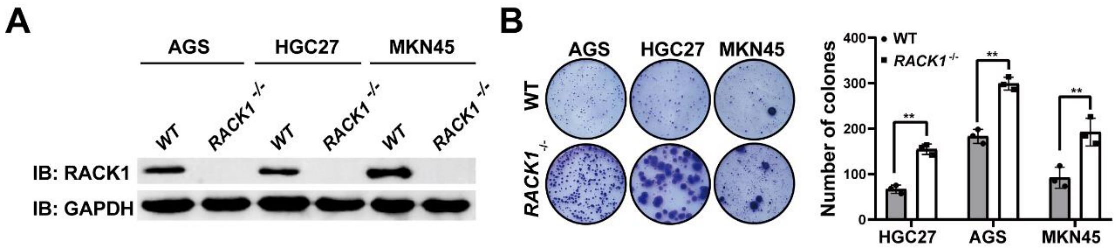

Fig. S3 a RACK1 knockout efficiencies were determined by western blotting. **b** colony formation assay in Wild-type and $RACK1^{-/-}$ HGC27, AGS and MKN45 cells. Student's t-test was used to examine statistical significance (Mean ± S.D., n = 3, $^{**}P < 0.01$, $^{*}P < 0.05$).
