# Supplementary material for: A novel UBE2T inhibitor suppresses Wnt/β-catenin signaling hyperactivation and gastric cancer progression by blocking RACK1 ubiquitination
Source: Oncogene. 2020 Dec 15;40(5):1027–42. doi: 10.1038/s41388-020-01572-w (PMC7862066; doi:10.1038/s41388-020-01572-w)
Supplement: Supplementary file 4 — Fig. S4 [file 41388_2020_1572_MOESM4_ESM.pdf]

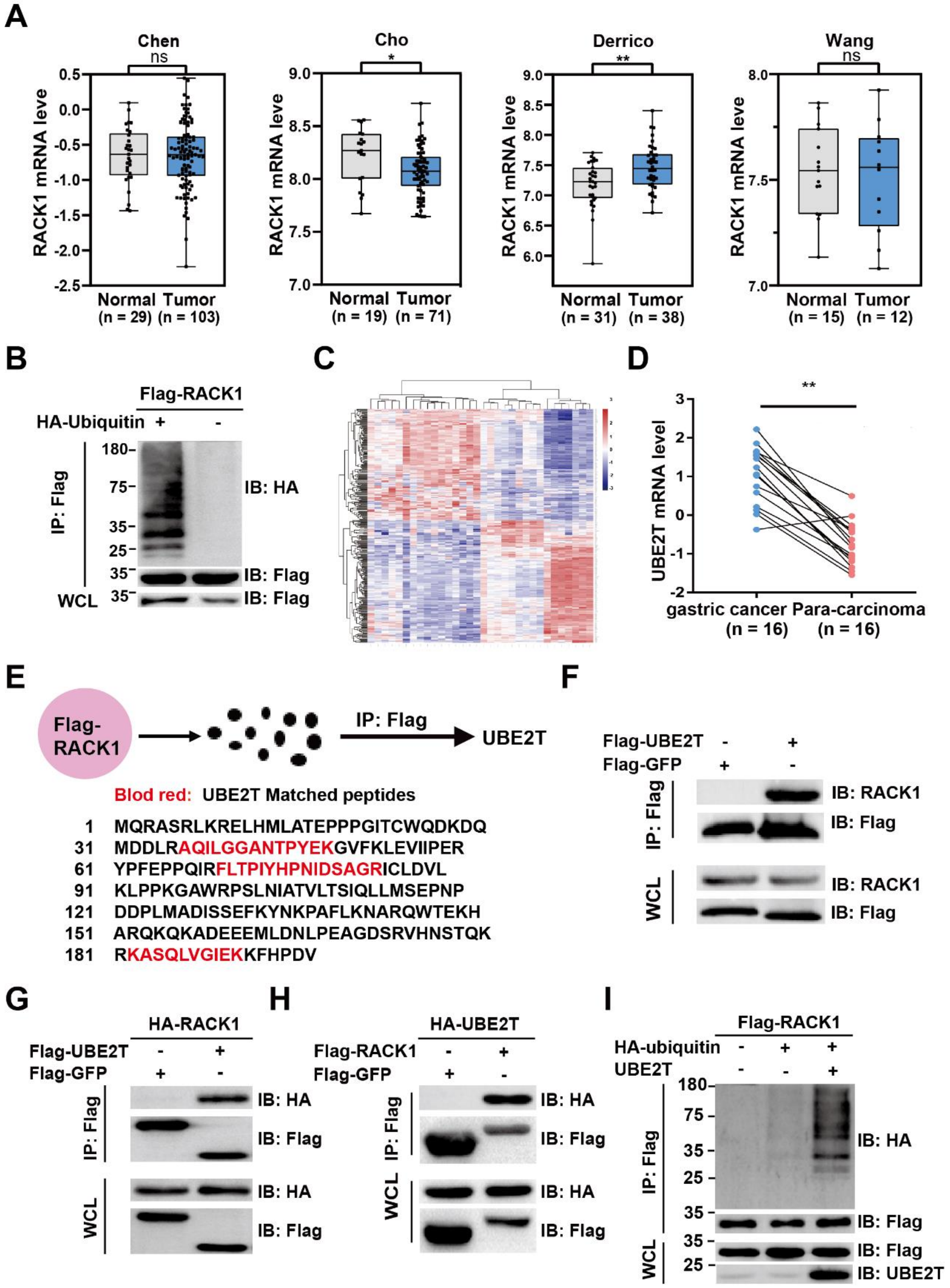

Fig. S4 **a** Data mining for RACK1 transcription using datasets from the Oncomine database. **b** Flag-tagged RACK1 and HA-tagged Ub Plasmids were co-transfected into HEK 293T cells for 24h, followed by cell lysate preparation and IP assay with anti-Flag beads followed by immunoblotting with indicated antibodies. IP: immunoprecipitates; WCL: whole cell lysates. **c** Heat map showing gene mRNA

expression profiles of 16 matched GC and normal gastric tissue samples. Red: up-regulated; Green: down-regulated. **d** Relative expression of UBE2T from tissue microarrays of 16 matched GC and para-carcinoma gastric tissue samples. **e** The identified peptides of UBE2T were marked in red. **f** A plasmid expressing Flag-tagged UBE2T or a plasmid of GFP was transfected into HGC-27 cells. Sixteen hours after transfection, cells were treated with MG132 for 8h (10uM). Cell lysates were analyzed by immunoprecipitation with anti-Flag and western immunoblotting with indicated antibodies. **g, h (g)** A plasmid expressing HA-tagged RACK1 was transfected into HEK 293T cells with a plasmid expressing Flag-tagged UBE2T or a plasmid of GFP. **(h)** A plasmid expressing HA-tagged UBE2T was transfected into HEK 293T cells with a plasmid expressing Flag-tagged RACK1 or a plasmid of GFP. Sixteen hours after transfection, cells were treated with MG132 for 8h (10uM). Cell lysates were analyzed by immunoprecipitation with anti-Flag and western immunoblotting with indicated antibodies. IP: immunoprecipitates; WCL: whole cell lysates. **i** HEK 293T cells were transiently transfected with plasmids encoding Flag-tagged RACK1 and UBE2T, along with plasmids encoding HA-tagged ubiquitin. Sixteen hours after transfection, cells were treated with MG132 for 8h (10μM). Cell lysates were degenerated and then analyzed by immunoprecipitation with anti-Flag and western immunoblotting with indicated antibodies.
