# Supplementary material for: A novel UBE2T inhibitor suppresses Wnt/β-catenin signaling hyperactivation and gastric cancer progression by blocking RACK1 ubiquitination
Source: Oncogene. 2020 Dec 15;40(5):1027–42. doi: 10.1038/s41388-020-01572-w (PMC7862066; doi:10.1038/s41388-020-01572-w)
Supplement: Supplementary file 5 — Fig. S5 [file 41388_2020_1572_MOESM5_ESM.pdf]

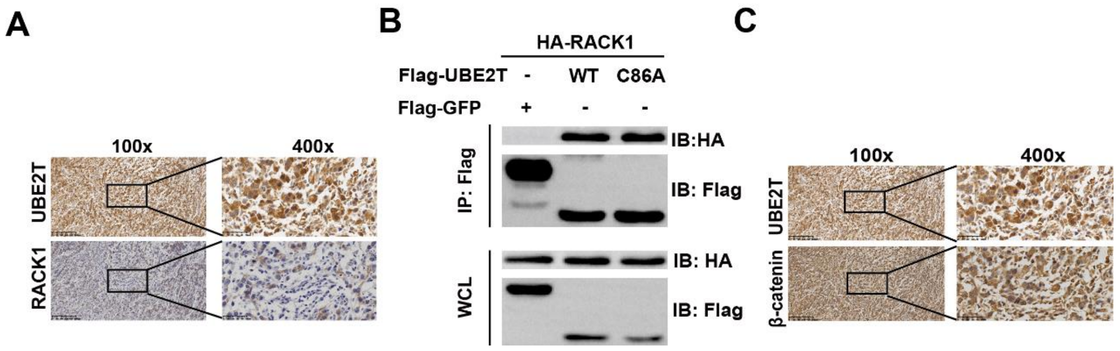

Fig. S5 a The Representative immunohistochemical images of UBE2T and RACK1 in gastric cancer tissues. Scale bar, 40 μm. **b** A plasmid expressing Flag-tagged wild-type UBE2T, UBE2T^{C86A} or GFP were transfected into HEK 293T cells with a plasmid expressing HA-tagged RACK1. Sixteen hours after transfection, cells were treated with MG132 for 8h (10uM). Cell lysates were analyzed by immunoprecipitation with anti-Flag and western immunoblotting with indicated antibodies. **c** The Representative immunohistochemical images of UBE2T and β-catenin in gastric cancer tissues. Scale bar, 40 μm.
