# Supplementary material for: A novel UBE2T inhibitor suppresses Wnt/β-catenin signaling hyperactivation and gastric cancer progression by blocking RACK1 ubiquitination
Source: Oncogene. 2020 Dec 15;40(5):1027–42. doi: 10.1038/s41388-020-01572-w (PMC7862066; doi:10.1038/s41388-020-01572-w)
Supplement: Supplementary file 6 — Fig. S6 [file 41388_2020_1572_MOESM6_ESM.pdf]

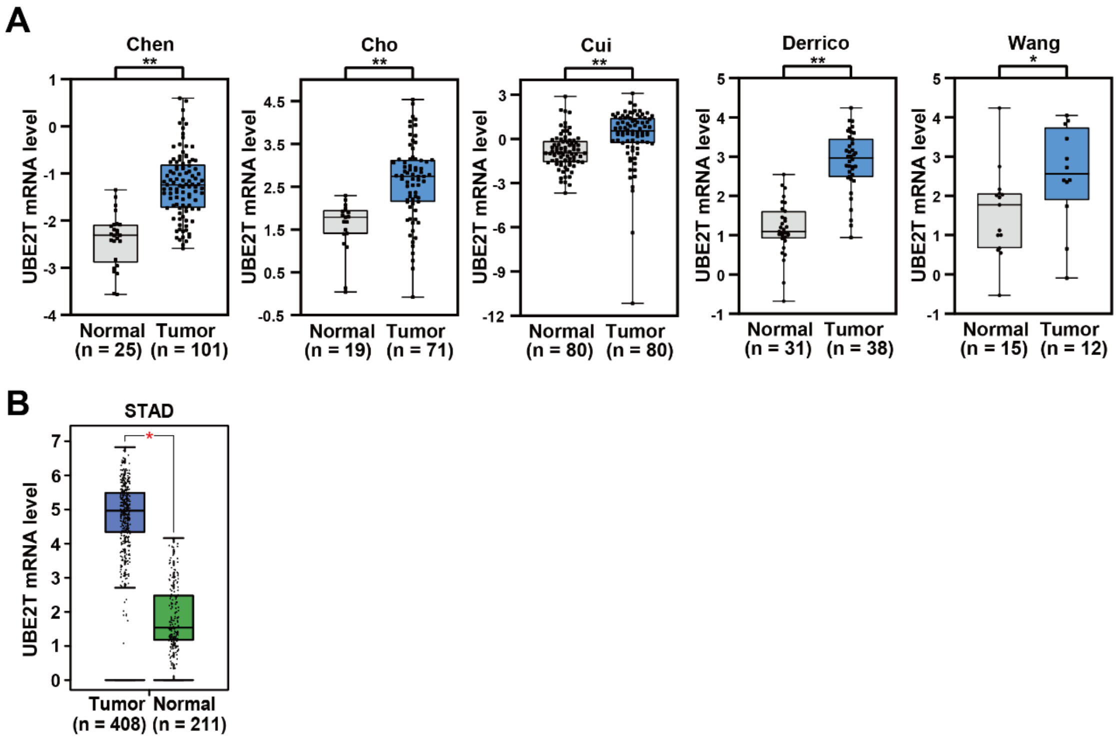

Fig. S6 a Data mining for UBE2T transcription using datasets from the Oncomine database. Five independent datasets consistently showed a significantly elevated level of UBE2T mRNA in gastric cancer tissues compared with normal gastric tissues. **b** UBE2T mRNA expression in Stomach adenocarcinoma (STAD) tissues and normal tissues were analyzed by Gene Expression Profiling Interactive Analysis (GEPIA) database.
