# Supplementary material for: A novel UBE2T inhibitor suppresses Wnt/β-catenin signaling hyperactivation and gastric cancer progression by blocking RACK1 ubiquitination
Source: Oncogene. 2020 Dec 15;40(5):1027–42. doi: 10.1038/s41388-020-01572-w (PMC7862066; doi:10.1038/s41388-020-01572-w)
Supplement: Supplementary file 8 — Fig. S8 [file 41388_2020_1572_MOESM8_ESM.pdf]

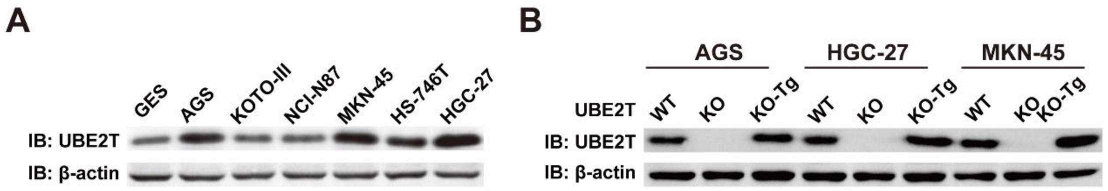

Fig. S8 a The expression of UBE2T in six GC cell lines was examined by western blots compared with human gastric epithelial cell line (GES-1). **b** UBE2T knockout efficiencies were determined by western blotting.
