# Supplementary material for: A novel UBE2T inhibitor suppresses Wnt/β-catenin signaling hyperactivation and gastric cancer progression by blocking RACK1 ubiquitination
Source: Oncogene. 2020 Dec 15;40(5):1027–42. doi: 10.1038/s41388-020-01572-w (PMC7862066; doi:10.1038/s41388-020-01572-w)
Supplement: Supplementary file 9 — Fig. S9 [file 41388_2020_1572_MOESM9_ESM.pdf]

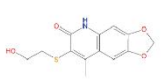

compound code:
AJ-292/41694981
docking score: -8.126
mol MW: 279.31

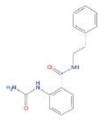

compound code:
AQ-360/42595630
docking score: -7.955
mol MW: 283.329

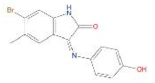

compound code:
AG-690/34979062
docking score: -7.903
mol MW: 331.168

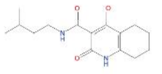

compound code:
AG-690/40721102
docking score: -7.876
mol MW: 278.35

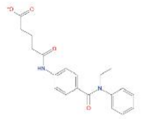

compound code:
AN-329/43449094
docking score: -7.799
mol MW: 354.405

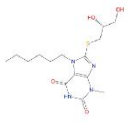

compound code:
AG-690/12244866
docking score: -7.789
mol MW: 356.439

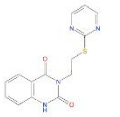

compound code:
AN-919/40998090
docking score: -7.641
mol MW: 300.334

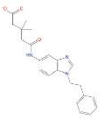

compound code:
AO-080/43378936
docking score: -7.592
mol MW: 379.458

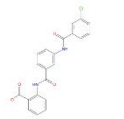

compound code:
AK-918/13161073
docking score: -7.571
mol MW: 394.813

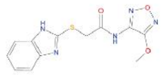

compound code:
AK-968/37053085
docking score: -7.564
mol MW: 305.311

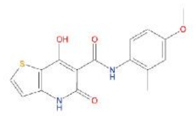

compound code: P163-0141
docking score: -8.49
mol MW: 330.358

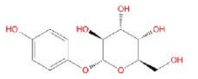

compound code: N034-0001
docking score: -8.441
mol MW: 272.254

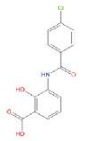

compound code: 8016-7035
docking score: -8.371
mol MW: 291.69

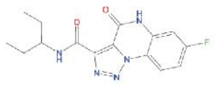

compound code: P516-0771
docking score: -8.294
mol MW: 317.322

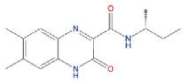

compound code: Z601-6470
docking score: -8.067
mol MW: 273.334

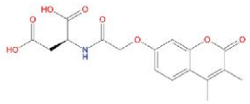

compound code: D715-2871
docking score: -8.049
mol MW: 363.323

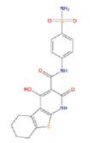

compound code: M435-1279
docking score: -8.028
mol MW: 419.469

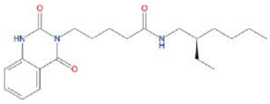

compound code: K221-2939
docking score: -8.017
mol MW: 373.494

Fig. S9 The selected top-scored 18 small molecule compounds with the highest affinity to UBE2T from Chemdiv and Specs compounds libraries by using Schrödinger software.
