# Supplementary material for: A novel UBE2T inhibitor suppresses Wnt/β-catenin signaling hyperactivation and gastric cancer progression by blocking RACK1 ubiquitination
Source: Oncogene. 2020 Dec 15;40(5):1027–42. doi: 10.1038/s41388-020-01572-w (PMC7862066; doi:10.1038/s41388-020-01572-w)
Supplement: Supplementary file 10 — Fig. S10 [file 41388_2020_1572_MOESM10_ESM.pdf]

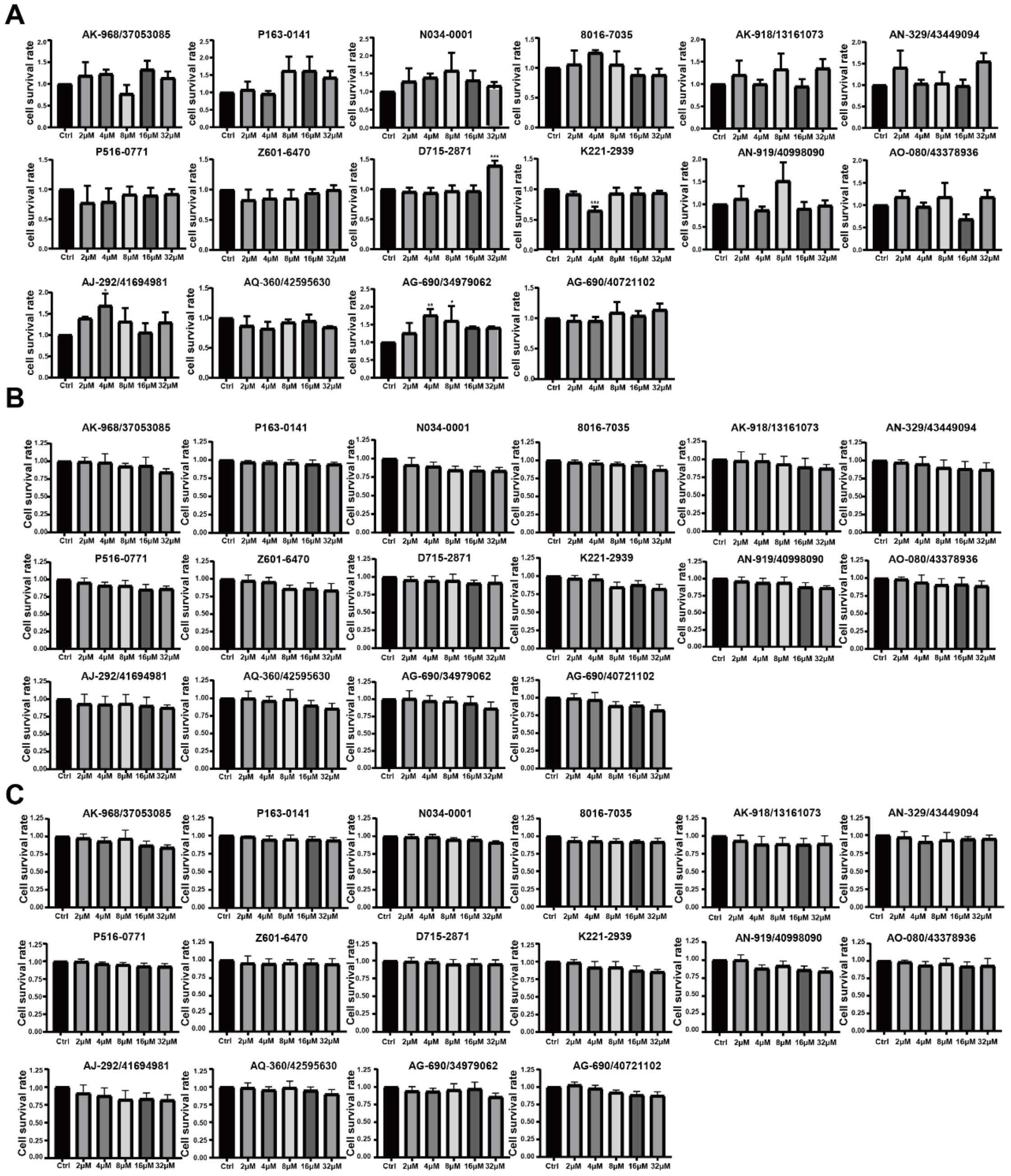

Fig. S10 a-c The effect of the other 16 top-scored small molecule compounds targeting UBE2T on HGC27 **(a)**, AGS **(b)** and MKN45 **(c)** cells growth. Cell viability was detected by 3-(4,5-dimethyl-2-thiazolyl)-2,5-diphenyl-2-H-tetrazolium bromide (MTT) assay. One-way analysis of variance (ANOVA) was used to examine statistical significance (Mean ± S.D., n = 6, $^{**}P < 0.01$, $^{*}P < 0.05$).
