# Supplementary material for: A novel UBE2T inhibitor suppresses Wnt/β-catenin signaling hyperactivation and gastric cancer progression by blocking RACK1 ubiquitination
Source: Oncogene. 2020 Dec 15;40(5):1027–42. doi: 10.1038/s41388-020-01572-w (PMC7862066; doi:10.1038/s41388-020-01572-w)
Supplement: Supplementary file 11 — Fig. S11 [file 41388_2020_1572_MOESM11_ESM.pdf]

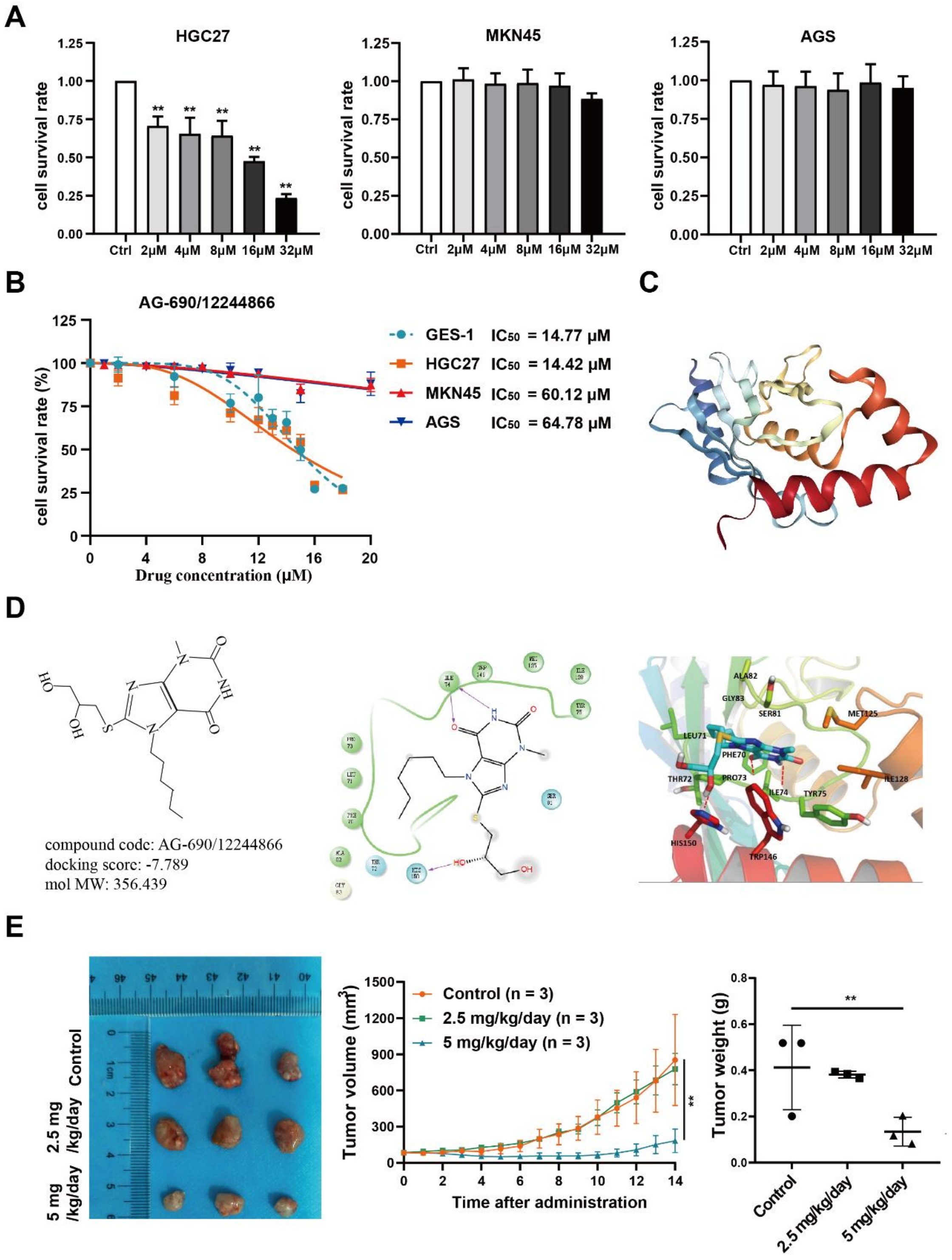

Fig. S11 a Cell survival rate of HGC27, AGS and MKN45 cells after 48h treated with AG-690/12244866. Cell viability was detected by 3-(4,5-dimethyl-2-thiazolyl)-2,5-diphenyl-2-H-tetrazolium bromide (MTT) assay. One-way analysis of variance (ANOVA) was used to examine statistical significance (Mean ± S.D., n = 6, $**P < 0.01$, $*P < 0.05$). **b** The effect of AG-690/12244866 on growth of HGC27, AGS,

MKN45 and GES-1 (as control), respectively. Cell viability was detected by 3-(4,5-dimethyl-2-thiazolyl)-2,5-diphenyl-2-H-tetrazolium bromide (MTT) assay. **c** UBE2T structure (PDB ID cod 1YH2). **d** A simulation snapshot of AG-690/12244866 with the allosteric site of UBE2T by molecular dynamics simulations. Sticks defined as the compounds and the active sites interacted with UBE2T, red thread dotted lines defined as hydrogen bonds between the compounds and UBE2T. For the compound, hydrogen: white, carbon: blue, oxygen: red, nitrogen: dark blue, and sulfur: yellow. **e** Tumor volume and weight of BALB/c mice treated with M435-1279 (intratumor injection 2.5mg/kg/day and 5mg/kg/day) and DMSO control (intratumor injection. 5mg/kg/day). One-way analysis of variance (ANOVA) was used to examine statistical significance (Mean ± S.D., n = 3, $^{**}P < 0.01$, $^{*}P < 0.05$).
