# Supplementary material for: A novel UBE2T inhibitor suppresses Wnt/β-catenin signaling hyperactivation and gastric cancer progression by blocking RACK1 ubiquitination
Source: Oncogene. 2020 Dec 15;40(5):1027–42. doi: 10.1038/s41388-020-01572-w (PMC7862066; doi:10.1038/s41388-020-01572-w)
Supplement: Supplementary file 12 — Table S1 [file 41388_2020_1572_MOESM12_ESM.docx]

**Table S1**. Correlation between RACK1 expression and the clinicopathologic characteristics of gastric cancer.

| Characteristics | RACK1 expression | | χ^2^ | *P*-value |
| --- | --- | --- | --- | --- |
|  | **High (n=37)** | **Low (n=118)** |  |  |
| Age |  |  |  |  |
| ≥ 55 | 17 | 73 | 2.931 | 0.087 |
| < 55 | 20 | 45 |  |  |
| Sex |  |  |  |  |
| Male | 26 | 82 | 0.008 | 0.928 |
| Female | 11 | 36 |  |  |
| Tumor location |  |  |  |  |
| Proximal stomach | 9 | 20 | 6.275 | 0.043 |
| Middle stomach | 16 | 32 |  |  |
| Distal stomach | 12 | 66 |  |  |
| Ki67 (%) |  |  |  |  |
| ≥ 70 | 11 | 31 | 0.056 | 0.813 |
| < 70 | 28 | 87 |  |  |
| Tumor size |  |  |  |  |
| ≥ 5 | 23 | 86 | 1.551 | 0.213 |
| < 5 | 14 | 32 |  |  |
| Histological type |  |  |  |  |
| Intestinal | 11 | 48 | 1.921 | 0.383 |
| Diffuse | 11 | 35 |  |  |
| Mixed | 15 | 35 |  |  |
| Depth of invasion |  |  |  |  |
| T1-2 | 20 | 21 | 19.356 | < 0.0001 |
| T3 | 2 | 18 |  |  |
| T4 | 15 | 79 |  |  |
| Lymph node |  |  |  |  |
| No-1 | 30 | 54 | 14.154 | < 0.0001 |
| N2-3 | 7 | 64 |  |  |
| Clinical stage |  |  |  |  |
| I | 18 | 18 | 19.134 | < 0.0001 |
| II | 8 | 28 |  |  |
| III | 11 | 68 |  |  |
| IV | 0 | 4 |  |  |
