# Supplementary material for: A novel UBE2T inhibitor suppresses Wnt/β-catenin signaling hyperactivation and gastric cancer progression by blocking RACK1 ubiquitination
Source: Oncogene. 2020 Dec 15;40(5):1027–42. doi: 10.1038/s41388-020-01572-w (PMC7862066; doi:10.1038/s41388-020-01572-w)
Supplement: Supplementary file 13 — Table S2 [file 41388_2020_1572_MOESM13_ESM.docx]

**Table S2.** Correlation between UBE2T expression and the clinicopathologic characteristics of gastric cancer patients.

| Characteristic | UBE2T expression | | χ^2^ | *P*-value |
| --- | --- | --- | --- | --- |
|  | **High (n=104)** | **Low (n=51)** |  |  |
| Age |  |  |  |  |
| ≥ 55 | 59 (56.7%) | 31 (60.8%) | 0.231 | 0.631 |
| < 55 | 45 (43.3%) | 20 (39.2%) |  |  |
| Sex |  |  |  |  |
| Male | 75 (72.1%) | 33 (64.7%) | 0.889 | 0.346 |
| Female | 29 (27.9%) | 18 (35.3%) |  |  |
| Tumor location |  |  |  |  |
| Proximal stomach | 19 (18.3%) | 10 (19.6%) | 2.966 | 0.227 |
| Middle stomach | 28 (26.9%) | 20 (39.2%) |  |  |
| Distal stomach | 57 (54.8%) | 21 (41.2%) |  |  |
| Ki67 (%) |  |  |  |  |
| ≥ 70 | 32 (30.8%) | 10 (19.6%) | 2.158 | 0.142 |
| < 70 | 72 (69.2%) | 41 (80.4%) |  |  |
| Tumor size |  |  |  |  |
| ≥ 5 | 81 (77.9%) | 28 (54.9%) | 8.661 | 0.003 |
| < 5 | 23 (22.1%) | 23 (45.1%) |  |  |
| Histological type |  |  |  |  |
| Intestinal | 40 (38.5%) | 19 (37.3%) | 0.546 | 0.761 |
| Diffuse | 29 (27.9%) | 17 (33.3%) |  |  |
| Mixed | 35 (33.6%) | 15 (29.4%) |  |  |
| Depth of invasion |  |  |  |  |
| T1-2 | 18 (17.3%) | 23 (45.1%) | 20.694 | < 0.0001 |
| T3 | 10 (9.6%) | 10 (19.6%) |  |  |
| T4 | 76 (73.1%) | 18 (35.3%) |  |  |
| Lymph node |  |  |  |  |
| No-1 | 43 (41.3%) | 41 (80.4%) | 59.93 | < 0.0001 |
| N2-3 | 61 (58.7%) | 10 (19.6%) |  |  |
| Clinical stage |  |  |  |  |
| I | 15 (14.4%) | 21 (41.2%) | 28.501 | < 0.0001 |
| II | 18 (17.3%) | 18 (35.3%) |  |  |
| III | 67 (64.4%) | 12 (23.5) |  |  |
| IV | 4 (3.9%) | 0 (0) |  |  |
