# Supplementary material for: A novel UBE2T inhibitor suppresses Wnt/β-catenin signaling hyperactivation and gastric cancer progression by blocking RACK1 ubiquitination
Source: Oncogene. 2020 Dec 15;40(5):1027–42. doi: 10.1038/s41388-020-01572-w (PMC7862066; doi:10.1038/s41388-020-01572-w)
Supplement: Supplementary file 14 — Table S3 [file 41388_2020_1572_MOESM14_ESM.docx]

**Table S3.** The corresponding vector, and primer sequence of plasmids in this study.

| Plasmid | Vector | Primer sequence |
| --- | --- | --- |
| Lent-crispri/cas9 -UBE2T-puro | Lent- crispri/cas9 -puro | FW: TGTGGAAAGGACGAAACACCGGGAGTGAGAAATCGGATCTG GTTTTAGAGCTAGAAATAGCA  RV: TGCTATTTCTAGCTCTAAAACCAGATCCGATTTCTCACTCC CGGTGTTTCGTCCTTTCCACA |
| Lent-crispri/cas9 -RACK1-puro | Lent- crispri/cas9 -puro | FW: TGTGGAAAGGACGAAACACCGGCCCACAAATCGCCTCGTGG GTTTTAGAGCTAGAAATAGCA  RV: TGCTATTTCTAGCTCTAAAACCCACGAGGCGATTTGTGGGC CGGTGTTTCGTCCTTTCCACA |
| Lent-CMV-Flag-UBE2T-puro | Lent-CMV-Flag-puro | FW: TACAAGGACGATGACGATAAGCAGAGAGCTTCACGTCTGAAG  RV: GTCCATGAATTCTAGACCCTACTAAACATCAGGATGAAATTT |
| Lent-CMV-Flag-UBE2T-Hygl | Lent-CMV-Flag-Hygl | FW: TACAAGGACGATGACGATAAGCAGAGAGCTTCACGTCTGAAG  RV: GTCATCGTCCTTGTAGTCCATCTAAACATCAGGATGAAATTTCTT |
| Lent-CMV-Flag-RACK1-Hygl | Lent-CMV-Flag-Hygl | FW: TACAAGGACGATGACGATAAGACTGAGCAGATGACCCTTCGT  RV: GTCCATGAATTCTAGACCCTACTAGCGTGTGCCAATGGTCACCTG |
| PRK5-flag-UBE2T | PRK5-flag | FW: TACAAGGACGATGACGATAAG CAGAGAGCTTCACGTCTGAAG  RV: GCTTCCCGAATTCTAGACCCGCTAAACATCAGGATGAAATTTCTT |
| PRK5-flag-RACK1 | PRK5-flag | FW: TACAAGGACGATGACGATAAG ACTGAGCAGATGACCCTTCGT  RV: GCTTCCCGAATTCTAGACCCGCTAGCGTGTGCCAATGGTCACCTG |
| PRK5-flag-UBE2T-C86A | PRK5-flag | FW1: TACAAGGACGATGACGATAAGCAGAGAGCTTCACGTCTGAAG  RV1: AACATCCAGAGCAATCCTTCC AGCAGAATCAATGTTTGGATG  FW2: GGAAGGATTGCTCTGGATGTTCTCAAATTGCCACCAAAAGGT  RV2: GCTTCCCGAATTCTAGACCCGCTAAACATCAGGATGAAATTTCTT |
| PRK5-3HA-UBE2T | PRK5-3HA | FW: CCAGATTACGCTGCTCAGCGC CAGAGAGCTTCACGTCTGAAG  RV: CCAAGCTTCCCGAATTCTAGACTAAACATCAGGATGAAATTTCTT |
| PRK5-3HA-RACK1 | PRK5-3HA | FW: CCAGATTACGCTGCTCAGCGCACTGAGCAGATGACCCTT  RV: AAGCTTCCCGAATTCTAGACTAGCGTGTGCCAATGGTCAC |
| PRK5-UBE2T | PRK5 | FW: CAACTGCACCTCGGTTCTATCGCTAGCCACCATGCAGAGAGCTTCACGTCTG  RV: GCTTCCCGAATTCTAGACCCGCTA AACATCAGGATGAAATTTCTT |
| PRK5-flag-RACK1-De1 | PRK5-flag | FW: TACAAGGACGATGACGATAAGGAGACCAACTATGGAATTCCA  RV: ACAAGTTGGGCCATGGCGGCCCTAGCGTGTGCCAATGGTCAC |
| PRK5-flag-RACK1-De2 | PRK5-flag | FW1: TACAAGGACGATGACGATAAGATGACTGAGCAGATGACCCTT  RV1: TCGCCTCGTGGTGGTGCCCGTATCCCTGGTCAGTTTCCACAT  FW2: ATGTGGAAACTGACCAGGGATACGGGCACCACCACGAGGCGA  RV2: CAAGTTGGGCCATGGCGGCCCTAGCGTGTGCCAATGGTCAC |
| PRK5-flag-RACK1-De3 | PRK5-flag | FW1: TACAAGGACGATGACGATAAGATGACTGAGCAGATGACCCTT  RV1: TGGACAGTGTATTTGCACACTGTGAGATCCCAGAGGCGCAG  FW2: TGCGCCTCTGGGATCTCACAGTGTGCAAATACACTGTCCAG  RV2: CAAGTTGGGCCATGGCGGCCCTAGCGTGTGCCAATGGTCAC |
| PRK5-flag-RACK1-De4 | PRK5-flag | FW1: TACAAGGACGATGACGATAAGATGACTGAGCAGATGACCCTT  RV1: GTTGGTCTTCAGCTTGCAGTTACCCAGGGTATTCCATAGCTT  FW2: AAGCTATGGAATACCCTGGGTAACTGCAAGCTGAAGACCAAC  RV2: ACAAGTTGGGCCATGGCGGCCCTAGCGTGTGCCAATGGTCAC |
| PRK5-flag-RACK1-De5 | PRK5-flag | FW1: TACAAGGACGATGACGATAAGATGACTGAGCAGATGACCCTT  RV1: GTAAAGGTGTTTGCCTTCGTTAGCCAGGTTCCATACCTTGAC  FW2: TCAAGGTATGGAACCTGGCTAACGAAGGCAAACACCTTTAC  RV2: CAAGTTGGGCCATGGCGGCCCTAGCGTGTGCCAATGGTCAC |
| PRK5-flag-RACK1-De6 | PRK5-flag | FW1: TACAAGGACGATGACGATAAGATGACTGAGCAGATGACCCTT  RV1: ACTGATAACTTCTTGCTTCAGGAGATCCCATAACATGGCCTG  FW2: CAGGCCATGTTATGGGATCTCCTGAAGCAAGAAGTTATCAGT  RV2: CAAGTTGGGCCATGGCGGCCCTAGCGTGTGCCAATGGTCAC |
| PRK5-flag-RACK1-De7 | PRK5-flag | FW: TACAAGGACGATGACGATAAGATGACTGAGCAGATGACCCTT  RV: ACAAGTTGGGCCATGGCGGCCCTATTCATCTACAATGATCTTTCC |
| PRK5-flag-RACK1-K139R | PRK5-flag | FW1: TACAAGGACGATGACGATAAGATGACTGAGCAGATGACCCTT  RV1: GCTCTCATCCTGGACAGTGTAACGGCACACACCCAGGGTATT  FW2: AATACCCTGGGTGTGTGCCGTTACACTGTCCAGGATGAGAGC  RV2: ACAAGTTGGGCCATGGCGGCCCTAGCGTGTGCCAATGGTCAC |
| PRK5-flag-RACK1-K172R | PRK5-flag | FW1: TACAAGGACGATGACGATAAGATGACTGAGCAGATGACCCTT  RV1: CAGGTTCCATACCTTGACCAGACGGTCCCAGCCACAGGAGAC  FW2: GTCTCCTGTGGCTGGGACCGTCTGGTCAAGGTATGGAACCTG  RV2: ACAAGTTGGGCCATGGCGGCCCTAGCGTGTGCCAATGGTCAC |
| PRK5-flag-RACK1-K175R | PRK5-flag | FW1: TACAAGGACGATGACGATAAGATGACTGAGCAGATGACCCTT  RV1: GCAGTTAGCCAGGTTCCATACACGGACCAGCTTGTCCCAGCC  FW2: GGCTGGGACAAGCTGGTCCGTGTATGGAACCTGGCTAACTGC  RV2: ACAAGTTGGGCCATGGCGGCCCTAGCGTGTGCCAATGGTCAC |
| PRK5-flag-RACK1-K225R | PRK5-flag | FW1: TACAAGGACGATGACGATAAGATGACTGAGCAGATGACCCTT  RV1: ACCATCTAGCGTGTAAAGGTGACGGCCTTCGTTGAGATCCCA  FW2: GGGATCTCAACGAAGGCCGTCACCTTTACACGCTAGATGGT  RV2: CAAGTTGGGCCATGGCGGCCCTAGCGTGTGCCAATGGTCAC |
| PRK5-flag-RACK1-K257R | PRK5-flag | FW1: TACAAGGACGATGACGATAAGATGACTGAGCAGATGACCCTT  RV1: CTTTCCCTCTAAATCCCAGATACGGATGCTGGGGCCTGTGGC  FW2: CCACAGGCCCCAGCATCCGTATCTGGGATTTAGAGGGAAAG  RV2: CAAGTTGGGCCATGGCGGCCCTAGCGTGTGCCAATGGTCAC |
| PRK5-flag-RACK1-K264R | PRK5-flag | FW1: TACAAGGACGATGACGATAAGATGACTGAGCAGATGACCCTT  RV1: CTTCAGTTCATCTACAATGATACGTCCCTCTAAATCCCAGAT  FW2: ATCTGGGATTTAGAGGGACGTATCATTGTAGATGAACTGAAG  RV2: CAAGTTGGGCCATGGCGGCCCTAGCGTGTGCCAATGGTCAC |
| PRK5-flag-RACK1-K271R | PRK5-flag | FW1: TACAAGGACGATGACGATAAGATGACTGAGCAGATGACCCTT  RV1: GCTGGTACTGATAACTTCTTGACGCAGTTCATCTACAATGAT  FW2: ATCATTGTAGATGAACTGCGTCAAGAAGTTATCAGTACCAGC  RV2: CAAGTTGGGCCATGGCGGCCCTAGCGTGTGCCAATGGTCAC |
| PRK5-flag-RACK1-K280R | PRK5-flag | FW1: TACAAGGACGATGACGATAAGATGACTGAGCAGATGACCCTT  RV1: GTGCACTGGGGTGGTTCTGCACGGCTGCTGGTACTGATAAC  FW2: TTATCAGTACCAGCAGCCGTGCAGAACCACCCCAGTGCACC  RV2: CAAGTTGGGCCATGGCGGCCCTAGCGTGTGCCAATGGTCAC |
