# Supplementary material for: A novel UBE2T inhibitor suppresses Wnt/β-catenin signaling hyperactivation and gastric cancer progression by blocking RACK1 ubiquitination
Source: Oncogene. 2020 Dec 15;40(5):1027–42. doi: 10.1038/s41388-020-01572-w (PMC7862066; doi:10.1038/s41388-020-01572-w)
Supplement: Supplementary file 15 — Table S4 [file 41388_2020_1572_MOESM15_ESM.docx]

**Table S4.** The primer sequence of UBE2T, RACK1 and GAPDH.

| Gene | Primer sequence |
| --- | --- |
| UBE2T | FW: ATCCCTCAACATCGCAACTGT  RV: CAGCCTCTGGTAGATTATCAAGC |
| RACK1 | FW: AGCAGCAACCCTATCATCGTC  RV: TGAGATCCCATAACATGGCCT |
| GAPDH | FW: GCACCGTCAAGGCTGAGAAC  RV: TGGTGAAGACGCCAGTGGA |
