# Supplementary material for: A novel UBE2T inhibitor suppresses Wnt/β-catenin signaling hyperactivation and gastric cancer progression by blocking RACK1 ubiquitination
Source: Oncogene. 2020 Dec 15;40(5):1027–42. doi: 10.1038/s41388-020-01572-w (PMC7862066; doi:10.1038/s41388-020-01572-w)
Supplement: Supplementary file 16 — Table S5 [file 41388_2020_1572_MOESM16_ESM.docx]

**Table S5.** The information of antibody in this study.

| Antibodies | SOURCE | IDENTIFIER |
| --- | --- | --- |
| UBE2T | Abcam | ab154022 |
| RACK1 | Abcam | ab129084 |
| HA | Sigma | SAB1306169 |
| Flag | Sigma | SAB4301135 |
| β-catenin | Abcam | ab16051 |
| Ubiquitin | Abcam | ab7780 |
| Ki67 | Abcam | ab15580 |
| β-actin | Abcam | ab8227 |
| GAPDH | Abcam | ab181602 |
| Lamin B1 | Abcam | ab16048 |
