# Supplementary material for: A novel UBE2T inhibitor suppresses Wnt/β-catenin signaling hyperactivation and gastric cancer progression by blocking RACK1 ubiquitination
Source: Oncogene. 2020 Dec 15;40(5):1027–42. doi: 10.1038/s41388-020-01572-w (PMC7862066; doi:10.1038/s41388-020-01572-w)
Supplement: Supplementary file 17 — Supplementary Materials and Methods [file 41388_2020_1572_MOESM17_ESM.docx]

**Supplementary Materials and Methods**

**Data mining**

The gene expression data used from the Gene Expression Profiling Interactive Analysis (GEPIA) databases are publicly available. The gene expression profiling of 16 matched gastric cancer tissues and para-carcinoma tissues were conducted by gene microarray (GeneChip primeview human, 901838, Affymetrix).

The mRNA expression level of CTNNB1, RACK1 and UBE2T in gastric cancer tissues were analyzed using Oncomine (https://www.oncomine.org/). The log-transformed and normalized expression values of CTNNB1, RACK1 and UBE2T were extracted, analyzed, and read on Oncomine. A P value less than 0.05 was selected as a threshold to lower the false discovery rate.

**Plasmid Construction and transfection**

Plasmid were structured as the following steps: Firstly, the insert of gene was obtained via PCR of KOD enzyme system (KFX-101T, TOYOBO, Japan), specific restriction enzymes were used to cut the corresponding vector, and used DNA Gel Extraction Kit (DP103, Tiangen, China) to purify DNA. Secondly, connected the insert and vector via Gibson system. Thirdly, the products were transformed into Escherichia coli competent cells, then, coated plates, select monoclonal colony PCR. Finally, extracted plasmid and sequencing it (Plasmid Miniprep kits, Tiangen, China). The plasmid and corresponding vector, and primer sequence were listed in Supplementary Table 3.

For transfection, 1*10^6^ HEK-293T cells were seeded in 6-weel plate. After 16h, plasmid (1ug) were transfected by Lipofectamine 2000 (1ul).

**Virus Production and Infection**

HEK-293T cells (1*10^6^) were seeded in 6-weel plate, after 16h, transfected Lent-CRISPR-puro and the lentiviral packaging plasmids psPAX2 and pMD2.G in optiMEM medium using Lipofectamine 2000. Similarly, Lent-CMV and Pvsvg and PDD also were transfected. After 6h, DMEM with 10% fetal bovine serum was changed into plates. 36h after transfection, centrifugation was performed to remove the cell debris. And then, 1ml lentivirus and 2ul polybrene were used to infect cells.

**Quantitative Real-Time PCR**

Total RNA was extracted from tissues of 24 patients using TRIzol reagent (Invitrogen). Then, reverse-transcribed with the reverse transcription kit (RR037A, Takara Bio, Japan). The expression of mRNAs of UBE2T and RACK1 were determined with a LightCycler (RR390Q, Takara Bio, Japan) using the iTaq Universal TB Green Supermix (RR420Q, Takara Bio, Japan). The primer sequence of UBE2T, RACK1 and GAPDH were shown in Supplementary Table 4. And the results were analyzed by ΔΔCT methods.

**Colony Formation**

Colony formation was used to detect the ability of proliferation. 1000 cells were seeded in 35-mm dishes, the colonies were stained with 0.1% crystal violet after 14 days.

**Immunoblotting**

Cells (2*10^6^) were lysed using 60ul lysis buffer (1M Tris (PH7.4), 10% Triton-100, 1 M NaCl, 0.5M EDTA and dd H_2_O) in ice for 10 min. And then centrifuged at 12000rpm for 10 min to remove the debris and nuclei. And boiled for 5 minutes with SDS-buffer in 95°C. Finally, samples were used to perform western blot assay. The information of antibody used in this study were listed in Supplementary Table 5.

**Molecular docking**

Molecular docking method was used to screen small molecular ligands with possible binding modes on UBE2T, and then a scoring method was used to rank them in order to select the optimal one. The three-dimensional model of UBE2T full length sequence was built using X-ray diffraction in the outward-facing open conformation (PDB ID cod 1YH2) as a template. We used Discovery Studio Visualizer 2.5 (Accelrys, San Diego, CA, USA) to find the possible binding site (active site) from the 3D atomic coordinates of UBE2T, and then screened compounds in Chemdiv and SPECS small molecular compounds library through docking. The docking procedure was carried out for the unchanged conformation of the receptor and flexible ligand molecules. The protein–ligand complex structure in 3D (POSE) and estimated binding energy (SCORE) were requested by inspection of the –Cdocker energy and –Cdocker interaction energy. Eighteen top-scored SMCs were selected based on the binding affinity.

**Microscale thermophoresis**

MST experiments were conducted on a Monolith NT.115 system (NanoTemper Technologies GmbH, Germany), which were used to quantify the interaction between UBE2T and RACK1, the interaction between UBE2T and M435-1279, respectively. UBE2T was labeled with the manufacturer’s labeling kits (Monolith^TM^ RED-NHS, NanoTemper Technologies GmbH, Germany), The UBE2T solutions were prepared in 130 mM NaHCO_3_ (pH 8.3), containing 50 mM NaCl, and RACK1 solutions were prepared in 10 mM PBS (pH 7.4). During the measurement of UBE2T-RACK interaction, the concentration of UBE2T is 200 nM, RACK1 is titrated from 61 nM to 2 *μ*M, the mixed solution of UBE2T and RACK1 containing 0.05% Tween 20. During the measurement of UBE2T with M435-1279, the concentration of labeled UBE2T is 100 nM, Unlabeled M435-1279 was prepared in 10 mM PBS dimethyl sulfoxide (DMSO), with the final concentrations of M435-1279 ranging from 31nM to 0.5 *μ*M. the samples were added to the monolith capillaries (MO L022, NanoTemper Technologies) and subsequently subjected to MST analysis. The values obtained were normalized and plotted against the RACK1 and M435-1279 concentration, respectively. The dissociation constant was then determined using a single-site model to fit the curve.

**Immunoprecipitation and Co-immunoprecipitation**

For immunoprecipitation, Cells (2*10^7^) treated with MG132 (10uM, 8h, 133407-82-6, Abmole, USA) were washed with PBS and lysed in 1 ml lysis buffer. Lysis were centrifugated at 12000rpm for 5min to remove debris and nuclei. The lysis was incubated with 30μl Flag antibody gel beads (SLBZ1501, SIGMA, USA) at 4°C for 2h. And then washed the beads using lysis buffer 4 times, eluted the beads using 100 ul elution buffer (PH=3.5), neutralized it and identified by mass spectrometry.

For co-immunoprecipitation, Cells (1*10^7^) were lysed in 1 ml lysis buffer. 50 ul of this as whole cell lysis, and the rest of lysis was incubated with 20μl Flag antibody gel beads at 4°C for 2h. And then washed the beads using lysis buffer, eluted the beads using 50 ul elution buffer (PH=3.5), neutralized it and boiled for 5 minutes with SDS-buffer in 95°C. Finally, western blot was used to perform detection. For endogenous co-immunoprecipitation, firstly, the antibody binds to magnetic beads protein A/G (22202-20, BEAVER, China) at room temperature for 15min. Then, cells (2*10^7^) treated with MG132 were washed with PBS and lysed in 400 μL IP binding buffer (22202-20, BEAVER, China). Next, the cell lysates were immunoprecipitated overnight at 4 °C with magnetic beads protein A/G. Subsequently, the magnetic beads were washed four times with washing buffer (22202-20, BEAVER, China). Finally, the immune complexes were eluted using 1Х SDS Buffer for 5 min at 95 °C and analyzed by Western blot.

**Toxicity Prediction of compound**

PK properties such as absorption, distribution, metabolism, excretion and toxicity (ADMET) profiling of compounds were determined using the pkCSM ADMET descriptors algorithm protocol and the Discover Studio 4.0 (DS4.0) software package (Accelrys Software, Inc., San Diego, CA, United States).
